# Comparison of Signal-Analysis Techniques for Seismic Detection System for High-Speed Train Data: Effect of Bridge Structures

**DOI:** 10.3390/s20236805

**Published:** 2020-11-28

**Authors:** Mintaek Yoo, Jae Sang Moon

**Affiliations:** 1 Railroad Structure Research Team, Korea Railroad Research Institute, Uiwang 16105, Korea; thezes03@krri.re.kr; 2 Structural Department, Yooshin Engineering Corporation, Seoul 06252, Korea

**Keywords:** seismic detection technology, high-speed train, signal analysis, bridge response analysis

## Abstract

This study evaluated the earthquake warning system for high-speed trains using onboard accelerometers instead of expensive seismometers. Onboard accelerometers measure the train data additional to the earthquake acceleration. The measured earthquake acceleration could also be modified by railroad-supporting bridges. To develop the data analysis system, the virtual onboard data sets are synthesized using the train acceleration data and earthquake data. Not only the earthquake acceleration data but also the earthquake responses of bridges are used for the virtual onboard data synthesis. For the analysis of synthesized data, the short-time Fourier Transform (STFT), the wavelet transform (WT), and Wigner–Ville Distribution (WVD) methods have been compared. Results show that WVD provides the best detection performance while the computational costs are large.

## 1. Introduction

Korea used to be known as an earthquake-free zone. However, that notion changed with the Gyeongju earthquake of 2016 (M_L_ = 5.8) [1] and the Pohang earthquake of 2017 (M_L_ = 5.4) [2]. In case of an earthquake, an early-warning system that could quickly stop a train in the event of an earthquake could be very beneficial. The current KTX (Korea Train Express) earthquake warning system detects earthquakes using seismic acceleration sensors installed at railroad facilities. They notify engineers by wire so that they can decide what to do [3]. However, this system takes too long, and it is not widespread enough to alert those who might need it. Unfortunately, installing and maintaining seismic acceleration sensors at all railroad facilities and track locations would require unrealistically high costs and manpower. The most well-known earthquake early warning system for the train system is the Urgent Earthquake Detection and Alarm System (UrEDAS) of Shinkansen in Japan [4,5]. UrEDAS provides early warning based on P-waves approaching railways. P-waves are detected by seismic stations nearby railways. Although the UrEDAS could provide early warning, this system still requires high costs to install the seismic stations near railways. Therefore, the development of a new concept is required to minimize the costs and maximize the performance of the earthquake detection and warning system.

To meet these needs, several research projects on seismometer networks based on smart devices and the internet have been conducted. The Southern California Earthquake Center and Incorporated Research Institutions for Seismology have constituted a global earthquake monitoring network based on Micro-Electro-Mechanical Systems of various types of devices (Quake-Catcher Network, QCN: http://quakecatcher.net) [6,7,8]. The California Institute of Technology has developed Community Seismic Network (CSN: http://csn.caltech.edu), an earthquake monitoring network based on the USB seismometer [9,10,11,12,13,14,15]. MyShake (http://myshake.berkeley.edu), an earthquake monitoring network application based on smartphone sensors has been developed recently and used by more than 300,000 users. MyShake is known to detect earthquakes more accurately and provide faster alerts than the ShakeAlert (https://www.shakealert.org), an earthquake early warning system in the western United States [16,17,18].

Recently, the use of cheap onboard sensors instead of seismic acceleration sensors for the earthquake warning system has been suggested. Since KTX moves up to higher than 300 km/h which means that the train moves more than 100 m/s, the train could experience derailment even by small earthquakes. Therefore, the train must be stopped or slow down immediately after the occurrence of earthquakes. The objective of this study is to prevent the derailment of a train by providing real-time warning/train speed control as soon as the cheap onboard sensor detects earthquakes. Since the sensor must be installed at every train, the onboard sensor must be cheaper than general seismometers. Unlike the UrEDAS, the onboard sensor would not properly measure the P-wave, a very small wave with vertical motion. Therefore, the signal processing system which detects earthquakes in real-time from the lateral movement of the train is a must. Moon and Yoo analyzed vibration data taken from onboard acceleration sensors during train operations and evaluated their earthquake detection capability potential [19]. The limit of this study is that the study considers earthquake response from the general roadbed is measured to the sensor. However, the earthquake response would be different when a train crosses a bridge. As the sensor would measure the earthquake response of the bridge, the earthquake warning system must be able to detect earthquakes even with the train in on a bridge. For this reason, this study aims to provide a way to distinguish the earthquake response of a bridge from the train vibration. Additionally, onboard KTX sensor data are superimposed and distinguished from the history data of the Pohang, Gyeongju, and Hachinohe earthquakes. For the magnitudes of earthquake acceleration, those with return periods of 1000 and 2400 years are applied, and the seismic responses that could occur in trains on bridges are calculated via bridge modeling and structural response analysis. The superimposed acceleration time-history results are analyzed using the short-time Fourier transform (STFT), wavelet transform (WT), and Wigner–Ville distribution (WVD), which are representative time-frequency analysis methods. These are then compared with the train vibration measurement data. Based on this, the detection of the occurrence of seismic responses from train vibration data is well-analyzed. The earthquake warning system based on onboard sensors would provide an accurate warning with lower costs if the system performs appropriately.

## 2. Train Acceleration Data and Seismic-Response Data

### 2.1. Train Acceleration Data

**Figure 1:**
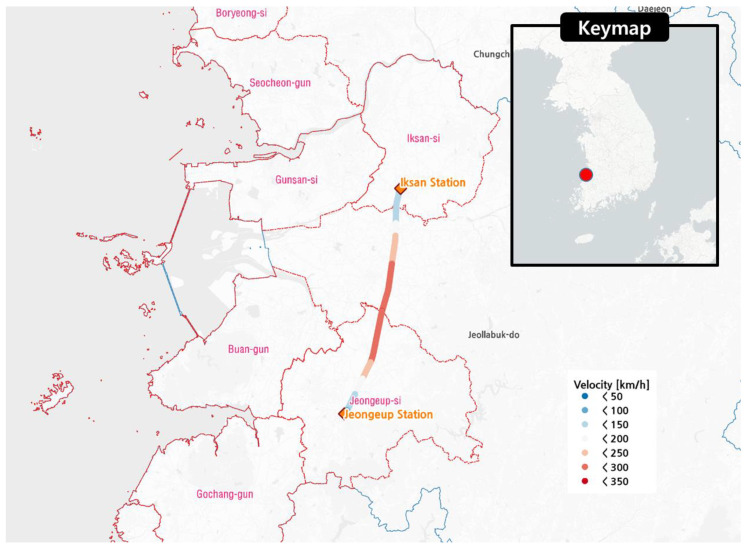
Travel route of the train for which train vibration data were collected.

**Figure 2:**
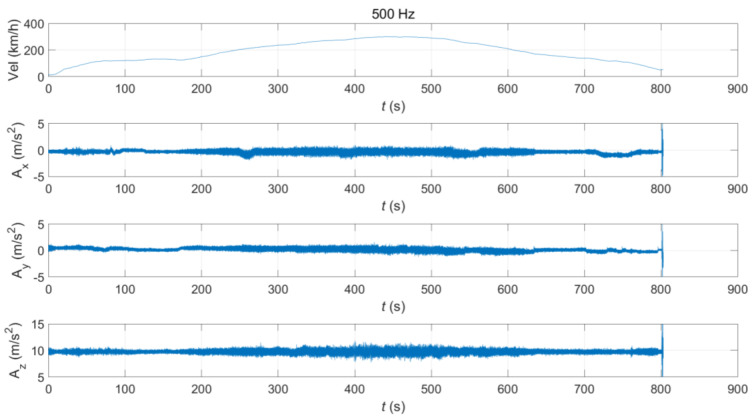
Korea Train Express (KTX) train vibration data measured by the onboard sensor.

This study used train vibration acceleration data measured by onboard sensors during the operation of KTX trains. The in-focus infrastructure was the Iksan–Gimje section, which has a length of approximately 41.8 km and an operation direction of south-southwest (Figure 1). The onboard sensor used in this study was a smartphone(android) sensor. The sampling frequency of this sensor was 500 Hz, and it was attached to a passenger car of the train. The accelerations were measured in the longitudinal (*x*), lateral (*y*), and vertical (*z*) directions and expressed as $A_{i}^{KTX}\left(t\right)\left(i=x,y,z\right)$. Figure 2 shows the velocity changes and the acceleration time history measured by the sensor in the operation section. Examining the operational characteristics along this section, the train continues after two accelerations, reaching a velocity of 300 km/h at around 450 s after starting, and it gradually decelerates after that.

### 2.2. Seismic-Response Data

#### 2.2.1. Earthquake Data

**Figure 3:**
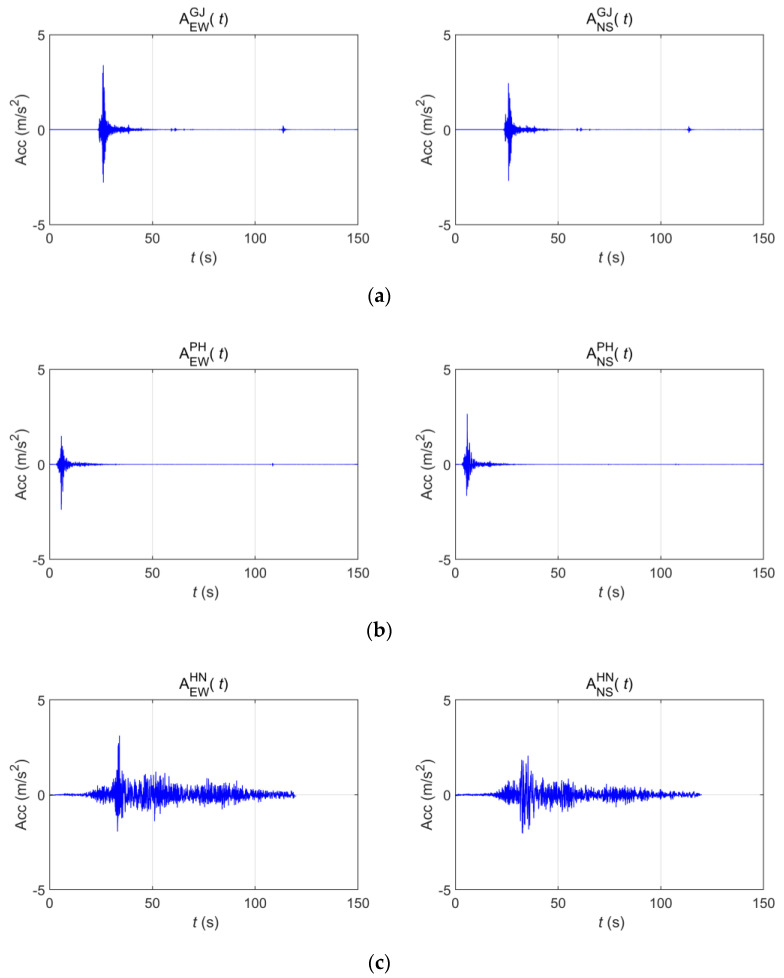
Measured acceleration time series of (**a**) Gyeongju (2016), (**b**) Pohang (2017), and (**c**) Hachinohe (1994).

**Figure 4:**
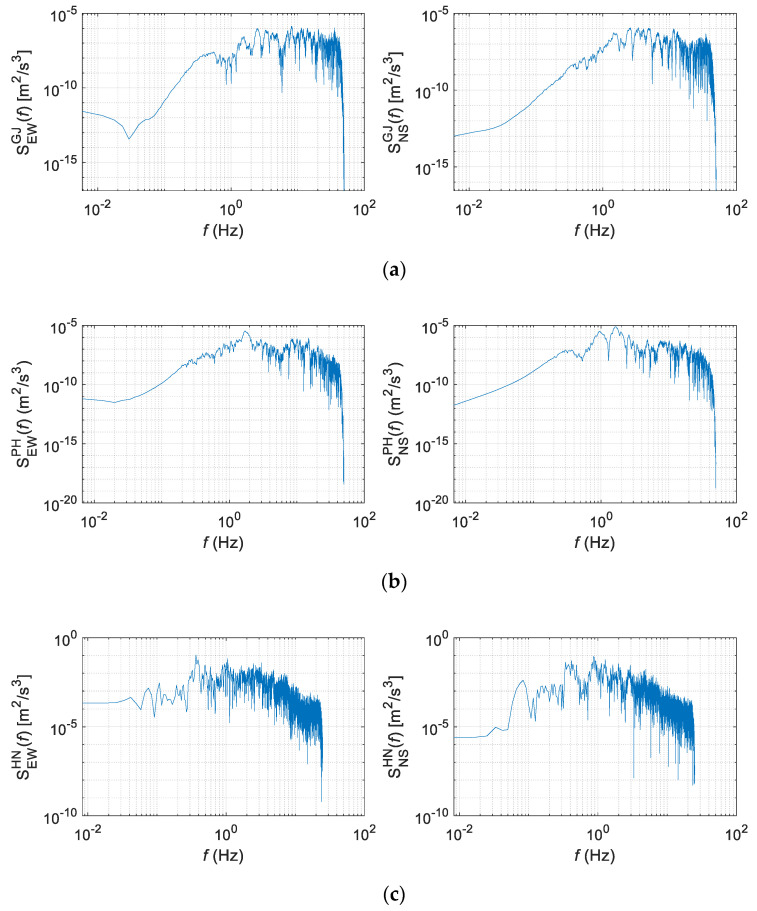
Power spectral densities (PSDs) of (**a**) Gyeongju (2016), (**b**) Pohang (2017), and (**c**) Hachinohe (1994).

The acceleration time-history data of three earthquakes were used. Table 1 provides basic information about each, and Figure 3 shows their acceleration time histories. The Gyeongju and Pohang earthquakes occurred in Korea, each having short-period seismic waves whose energies sharply decreased after the peak accelerations. The Hachinohe earthquake was a long-period seismic wave in which approximately 0.1 g of acceleration appeared continuously for approximately 90 s, even after the peak acceleration [20]. Figure 4 shows the power spectral density (PSD) of each wave. The Gyeongju and Pohang earthquakes showed low energy levels at 1 Hz or lower, but they exhibited a constant energy level, even below 0.1 Hz.

In this study, the three acceleration time history data were scaled to generate the maximum peak values of 0.154 and 0.22 g. These peak values correspond to return periods of 1000 and 2400 years in seismic design standards used for seismic design of railroad facilities in Korea [21].

#### 2.2.2. Bridge Seismic-Response Analysis

**Figure 5:**
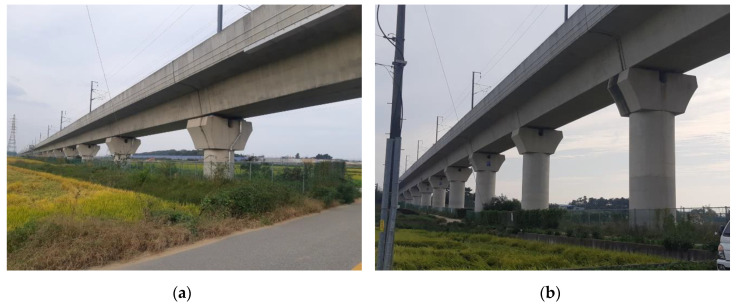
Images of (**a**) BR2 and (**b**) BR3.

This study selected BR2 and BR3 bridges, located 12.5 and 15.2 km, respectively, from the Iksan Station line to Jeongeup (Figure 5). The lateral seismic responses that can directly affect train operation were calculated for these two bridges. Both BR2 and BR3 are PSC box-girder-type isostatic bridges having spans of 30 m. They have the same girder and pier shapes, but their pier lengths are different.

**Figure 6:**
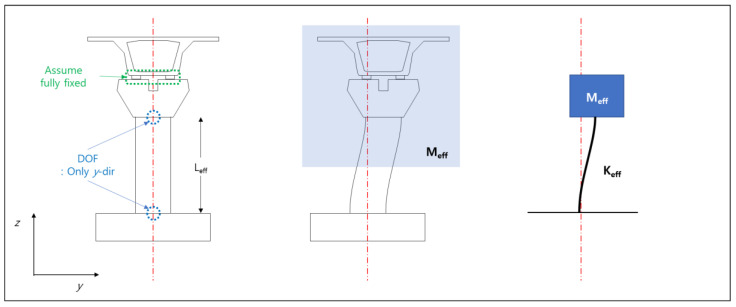
Diagram of bridge modeling (L_eff_, M_eff_, and K_eff_ denote length, mass, and stiffness of the SDOF model, respectively).

For the lateral behaviors of the two bridges, the seismic responses were calculated by constructing equivalent single-degree-of-freedom (SDOF) models. It was assumed that the lateral behaviors of the bridges were mainly affected by the structural characteristics of the piers and that the flexural stiffnesses were mainly affected by their column parts. Although the lateral response measured at the train could be affected by other factors, such as the characteristics of the bearing and foundation type, this study assumes that these factors are small. It was also assumed that the top-½ sections of the girders and piers acted as equivalent masses of the SDOF model (Figure 6). Regarding stiffness, the effective cross-sectional second moments were calculated by assuming linear behaviors for the materials and cross-sections. The equivalent stiffnesses were then calculated by assuming the effective lengths and behaviors in the piers of BR2 and BR3. Additionally, it was assumed that the pier tops only moved in the lateral direction (no rotation) and that plastic hinging did not occur at either end of the column. The damping ratios of the piers’ equivalent SDOF model were assumed to be 5% each, which is a value commonly used in domestic bridge design standards. Table 2 shows the mass, stiffness, and natural frequency of the equivalent SDOF model for each bridge. The natural frequencies for the lateral behaviors of BR2 and BR3 were 11.54 and 3.68 Hz, respectively. For the responses of SDOF models, the Newmark method was applied [22]. The structural damping is assumed to have the damping ratios mentioned above.

**Figure 7:**
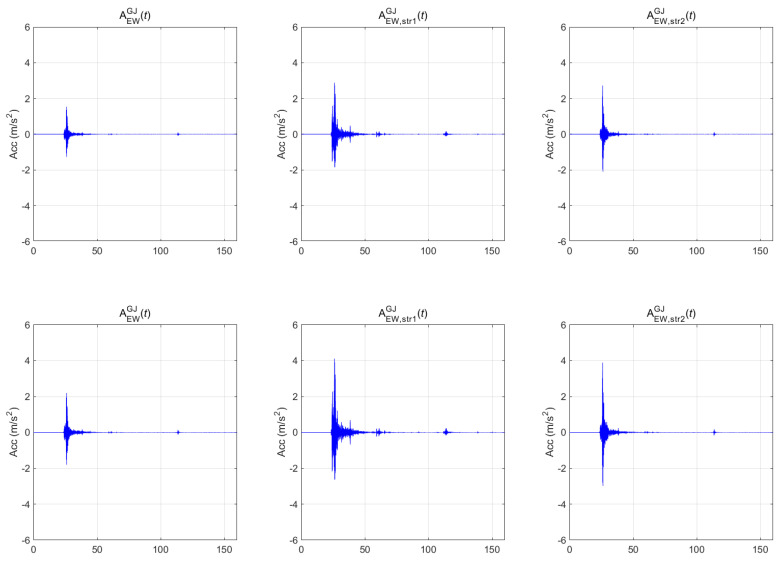
Bridge responses of the Gyeongju earthquake.

**Figure 8:**
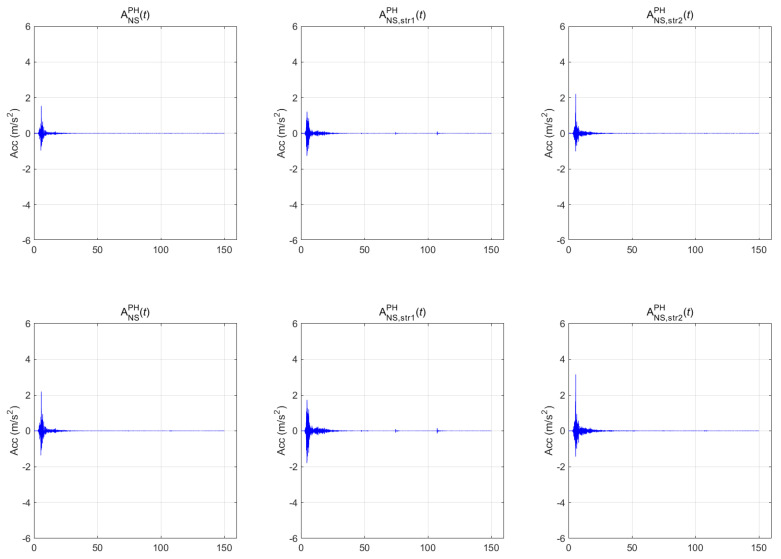
Bridge responses of the Pohang earthquake.

**Figure 9:**
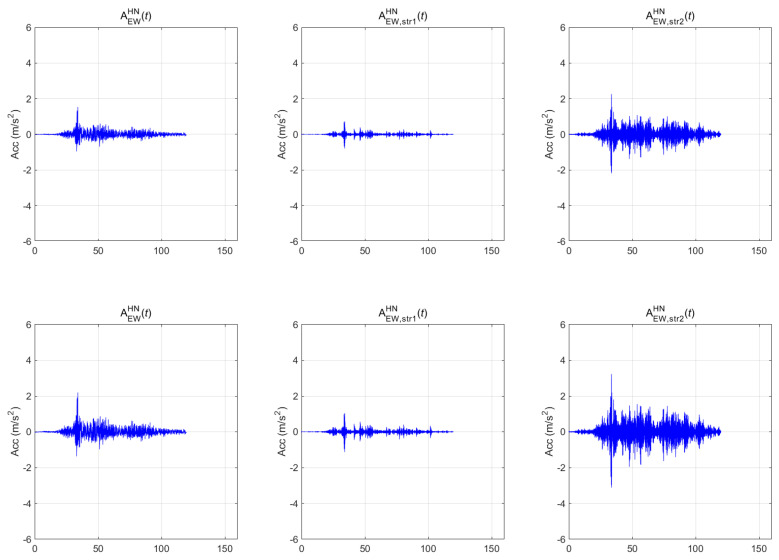
Bridge responses of the Hachinohe earthquake.

Figure 7, Figure 8 and Figure 9 show the seismic-response acceleration time histories of BR2 (subscripted as str1) and BR3 (subscripted as str2) for the three earthquakes, respectively. In each figure, the first, second, and third columns represent the scaled seismic waves, the lateral seismic response atop BR2, and the lateral seismic response atop BR3, respectively. As with the above-ground responses, the first- and second-row seismic waves were scaled to return periods of 1000 and 2400 years, respectively. In the case of the Gyeongju earthquake of Figure 7, the maximum accelerations were 0.388 g (1000 years) and 0.554 g (2400 years) for the BR2 lateral responses and 0.278 g (1000 years) and 0.396 g (2400 years) for the BR3 lateral responses. In the case of the Pohang earthquake of Figure 8, the maximum accelerations were 0.275 g (1000 years) and 0.393 g (2400 years) for the BR2 lateral responses and 0.225 g (1000 years) and 0.322 g (2400 years) for the BR3 lateral responses. In the case of the Hachinohe earthquake of Figure 9, the maximum accelerations were 0.155 g (1000 years) and 0.222 g (2400 years) for the BR2 lateral responses, and 0.231 g (1000 years) and 0.329 g (2400 years) for the BR3 lateral responses. Thus, the top responses were amplified in both bridges for the Gyeongju and Pohang earthquakes, whereas all peak accelerations decreased in BR2 for the Hachinohe earthquake.

## 3. Data Analysis

### 3.1. Data-Analysis Methods

To detect earthquakes from time-history data, an analysis method that can detect changes in their characteristics over time domain must be applied. STFT, WT, and WVD time-frequency analysis methods were evaluated for their applicability to earthquake detection.

#### 3.1.1. STFT

STFT verifies changes in frequency region over time-based on Fourier transform. It divides the given data into *n* windows and calculates the fast Fourier transform (FFT) and PSD for each window. The STFT-based PSD, $S_{A}\left(f,t\right)$, from the acceleration time history, $A\left(t\right)\;\left(0\leq t\leq T_{n}\right)$, is defined as follows:

(1) $$S_{A}\left(f,t\right)=\{\begin{array}{cc} S_{A,1}\left(f,t\right) & 0\leq t\leq T_{1}\\ \begin{array}{c} S_{A,2}\left(f,t\right)\\ \vdots \end{array} & \begin{array}{c} T_{1}\leq t\leq T_{2}\\ \vdots \end{array}\\ S_{A,n}\left(f,t\right) & T_{n-1}\leq t\leq T_{n} \end{array},$$

where $S_{A,i}\left(f,t\right)$ means that the PSD ($={\left|\mathrm{FFT}\left(A_{i}\left(t\right)\right)\right|}^{2}$) for the acceleration time history, $A_{i}\left(t\right)$ $\left(T_{i-1}\leq t\leq T_{i}\right)$, is in the *i*th window. STFT has been used to detect changes in the characteristics of time-history data per period in various other fields [23,24,25,26,27,28].

**Figure 10:**
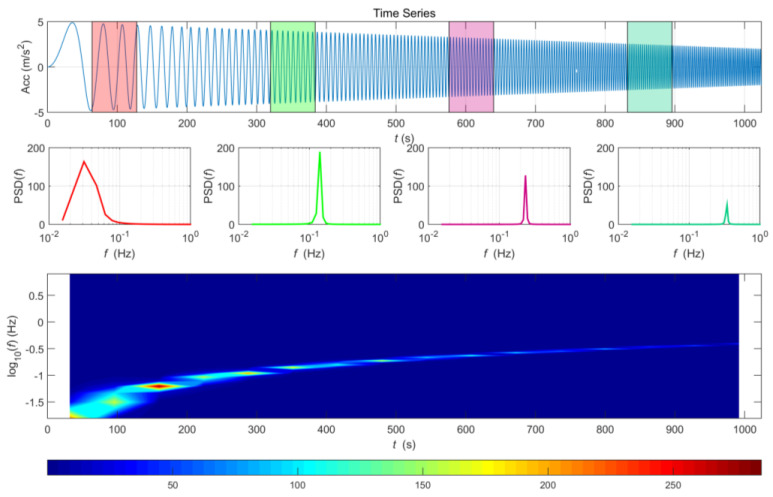
Short-time Fourier Transform (STFT) results for signals in which the amplitude and frequency change over time.

Figure 10 shows the STFT result for signals in which the amplitude and frequency change over time. The characteristics changing over time can be verified, unlike with general PSD. However, because the effects of resolution and noise in the STFT vary with the size of the divided sections, it is necessary to select an appropriate section size, depending on the data characteristics and points of interest. In this study, the window size was set to 256 points for earthquake analysis and 1024 sample points for acceleration data analysis of trains.

#### 3.1.2. WT

**Figure 11:**
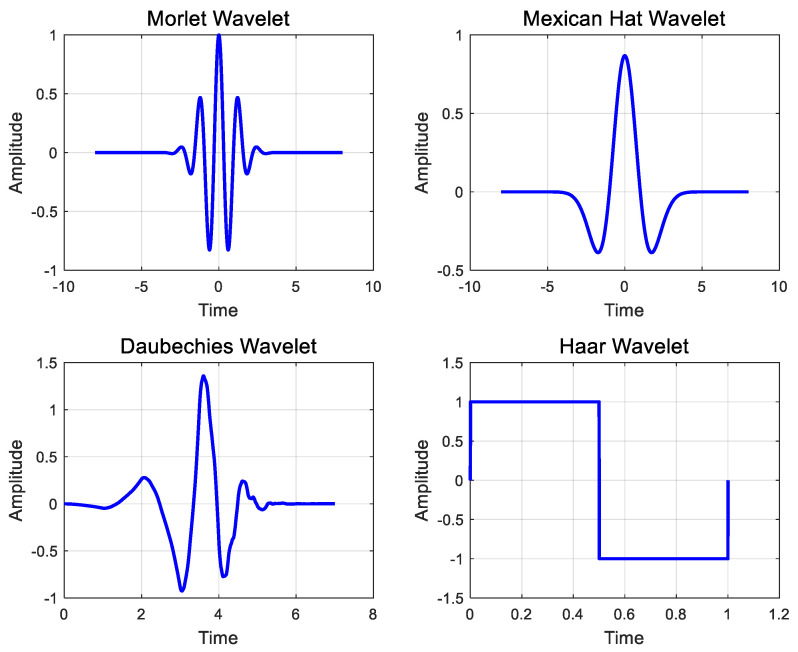
Example of mother wavelets.

WT reorganizes given signals to a set of specific ones similar to the Fourier transform [29,30,31]. In the case of a general Fourier transform, signals are reorganized into a combination of sine and cosine waves, whereas WT reorganizes the given signals using base signals (i.e., mother wavelets). Figure 11 shows the representative mother wavelets that can be used with WTs. Unlike sine and cosine waves, mother wavelets have fixed signal lengths and asymmetry. In the case of the general Fourier transform, the results only express changes in the frequency domain, because the transform uses sine and cosine waves of infinite lengths, assuming that given signals are repeated continuously. In contrast, WT uses finite mother wavelets, and the results express changes over time and frequency. This study expresses the WT of acceleration time history, A(t), as C(t,f). Similar to the Fourier transform, the energy distribution of the acceleration time history based on mother wavelets is ${\left|C\left(t,f\right)\right|}^{2}$. WT is known to capture the abrupt changes of signals better than STFT [32,33,34]. However, in the case of the WT, the resolution varies by the position in the time-frequency domain, compared with STFT. The higher the time resolution, the lower the frequency resolution, and vice versa. Furthermore, because the reorganization result varies by the mother wavelet, appropriate wavelets must be selected. In this study, the acceleration time history was analyzed using the Morlet wavelet.

#### 3.1.3. WVD

WVD was presented by Eugene Wigner [35], and the basic definition is as follows:

(2) $$W\left(t,f\right)=\int_{-\infty }^{\infty }A\left(t-\frac{\tau }{2}\right)A^{*}\left(t+\frac{\tau }{2}\right)e^{-2\pi i\tau f}d\tau .$$

**Figure 12:**
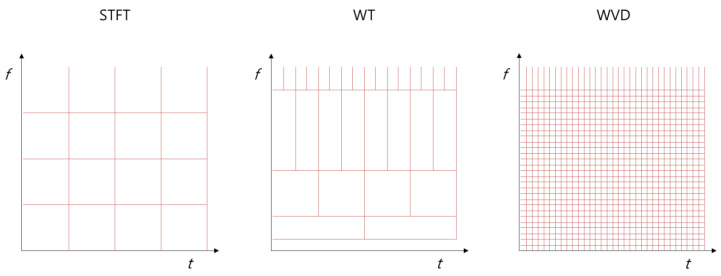
Diagram of the resolution of methods.

The WVD translates the time-dependent autocorrelation function of a process to the Fourier domain. The WVD is similar to power-spectral density, which is the Fourier transform of autocorrelation. If the acceleration time history, A(t), is a stationary process at all time intervals, the WVD at any *t* is *S*(*f*). When the WVD is integrated for all time and frequency domains, it becomes identical to the energy of time history, which is identical to the value of integrating the PSD for all frequency domains. One advantage of WVD is that it provides time-frequency analysis with high resolution. When a time history consisting of *N* samples is analyzed, the WVD produces a result value wherein the matrix size is 2*N* × N. As such, the WVD has the advantage of detecting specific events when they occur. Wigner–Ville distribution has been not only studied in earthquake detection [36,37,38,39] but also used in various areas for the detection of other nonstationary signals [40,41,42,43,44,45,46]. One disadvantage is that the calculation takes a long time because it implements high resolutions [47,48]. Figure 12 shows a diagram of the resolution in the time-frequency domain for each method. The WVD provides high resolution, unlike the other two methods. To evaluate its applicability to real-time detection of earthquakes in the present study, WVD is performed for windows consisting of 256 and 1024 sample points when analyzing earthquakes and train acceleration data, as with the STFT. Computational codes are written based on the MATLAB-based toolbox [49].

### 3.2. Data Characteristics

#### 3.2.1. Seismic Response of Bridges

**Figure 13:**
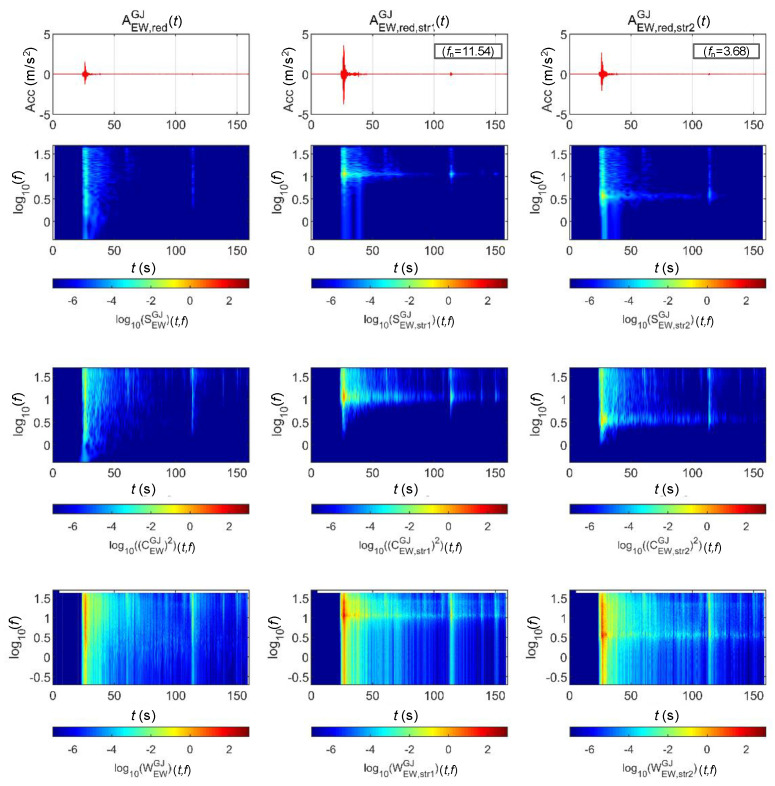
Analysis results of the Gyeongju earthquake data (scaled for 1000-year return period) and associated bridge responses.

**Figure 14:**
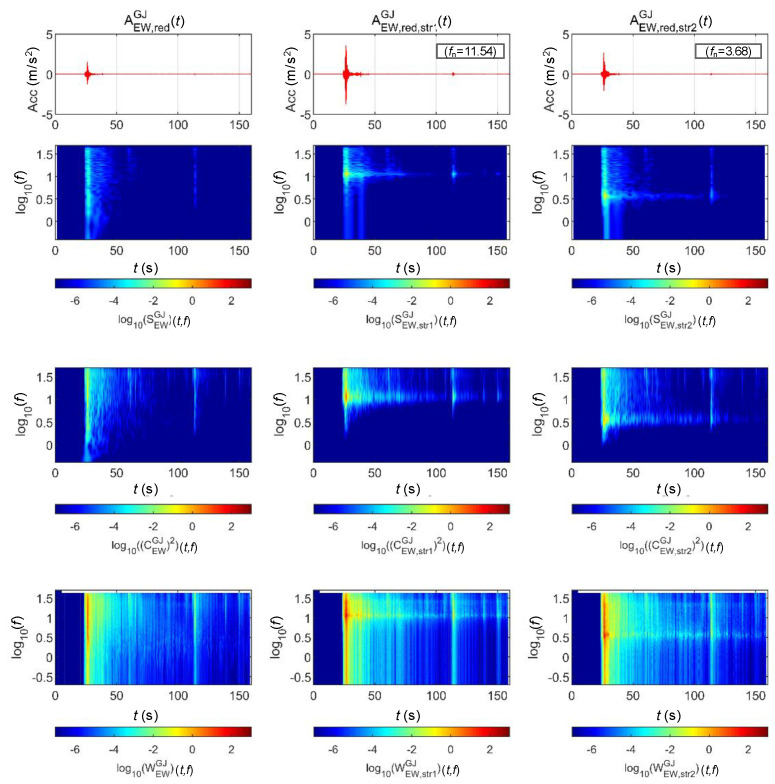
Analysis Results of the Gyeongju earthquake data (scaled for 2400-year return period) and associated bridge responses.

Figure 13 and Figure 14 show the STFT, WT, and WVD results for the accelerations and bridge responses of the Gyeongju earthquake adjusted to return periods of 1000 and 2400 years, respectively. The second and third columns show the analysis results of the top lateral responses of the BR2 and BR3 bridge piers, respectively.

First, the energy around the natural frequency of each bridge increased at approximately 25 s when the peak acceleration occurred as the signals passed through the bridge. This is because the bridge was modeled as a 1-DoF linear system. It can be seen that the energy around the natural frequency of the bridge also increased with the acceleration generated by the bridge at 110 s. The WVD clearly shows the change in energy over time compared to the other two methods.

**Figure 15:**
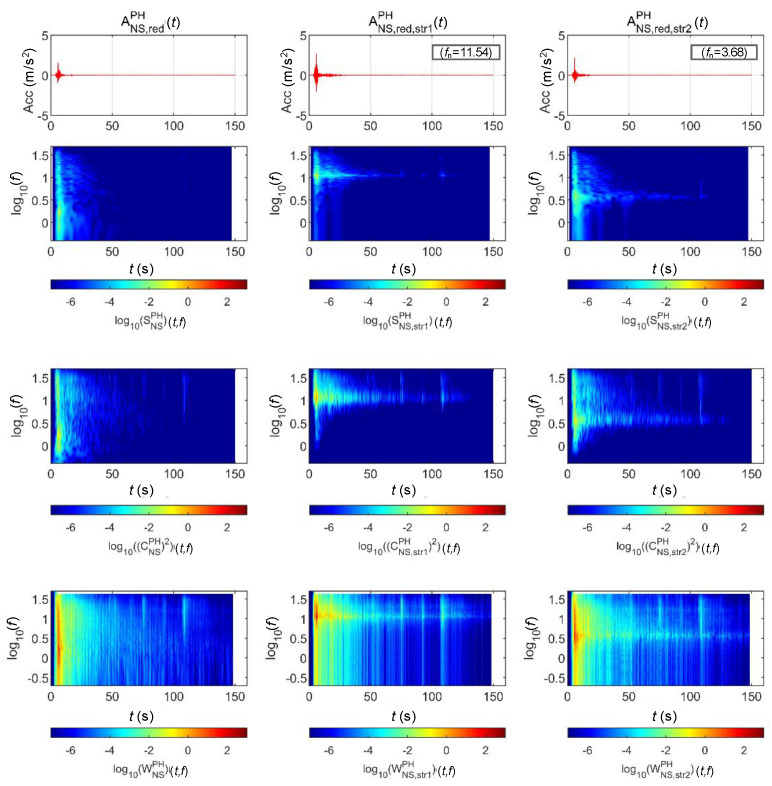
Analysis results of the Pohang earthquake data (scaled for 1000-year return period) and associated bridge response.

**Figure 16:**
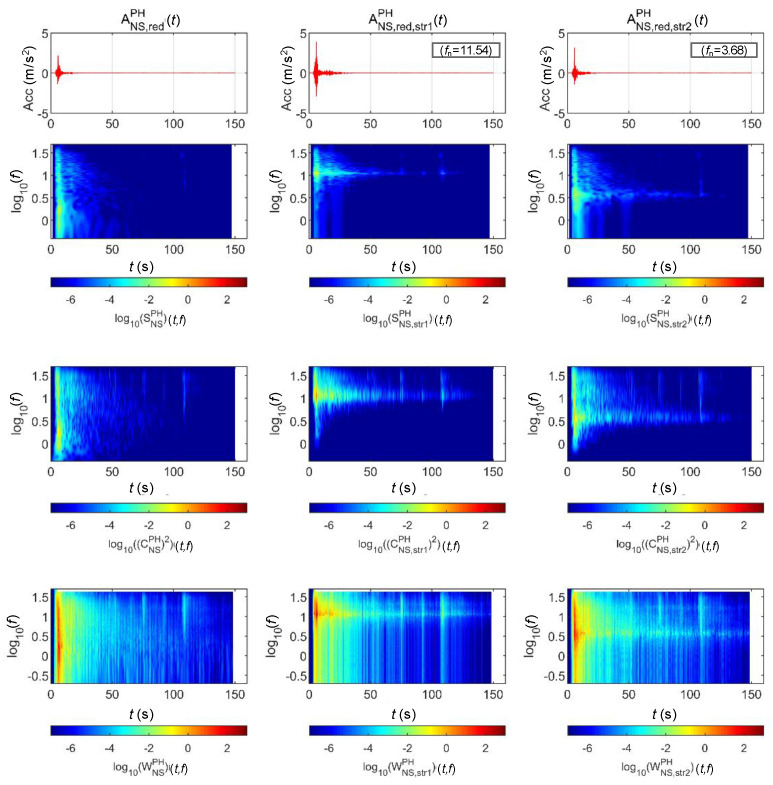
Analysis results of the Pohang earthquake data (scaled for 2400-year return period) and associated bridge response.

Figure 15 and Figure 16 show the STFT, WT, and WVD results for the accelerations and bridge responses of the Pohang earthquake adjusted to return periods of 1000 and 2400 years, respectively. First, the energy around the natural frequency of each bridge increased at around 10 s when the peak acceleration occurred as the signals passed through the bridge, and the energy decreased in the frequency domain outside the natural frequency. In the time domain after 10 s, only the energy in the region near the natural frequency of each bridge decreased relatively less with time, similar to Figure 13 and Figure 14. Lastly, in the Pohang earthquake and bridge responses, the WVD shows the changes in energy over time most dynamically, and the STFT shows the changes the least dramatically.

**Figure 17:**
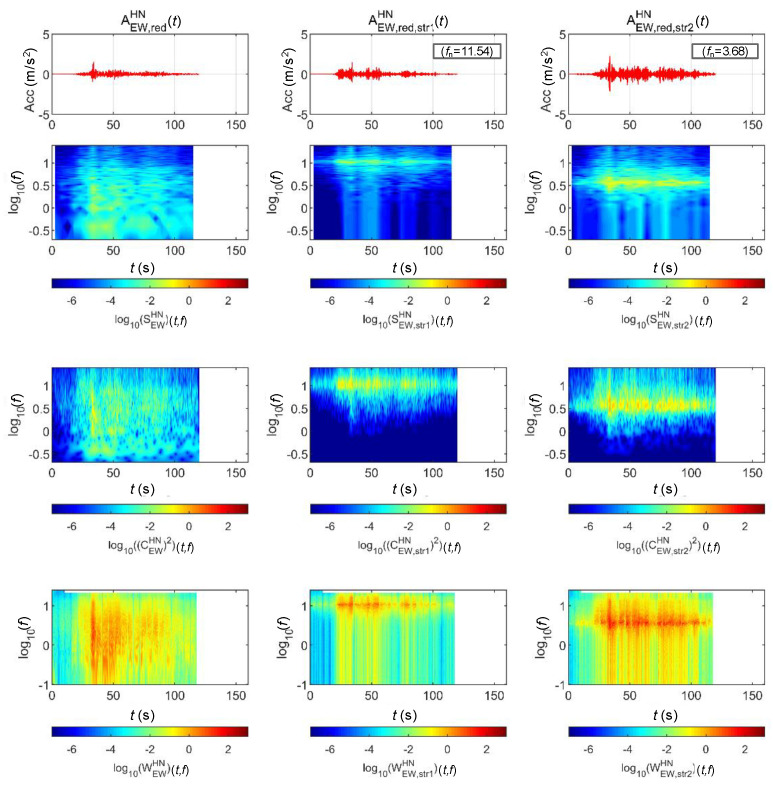
Analysis results of the Hachinohe earthquake data (scaled for 1000-year return period) and associated bridge response.

**Figure 18:**
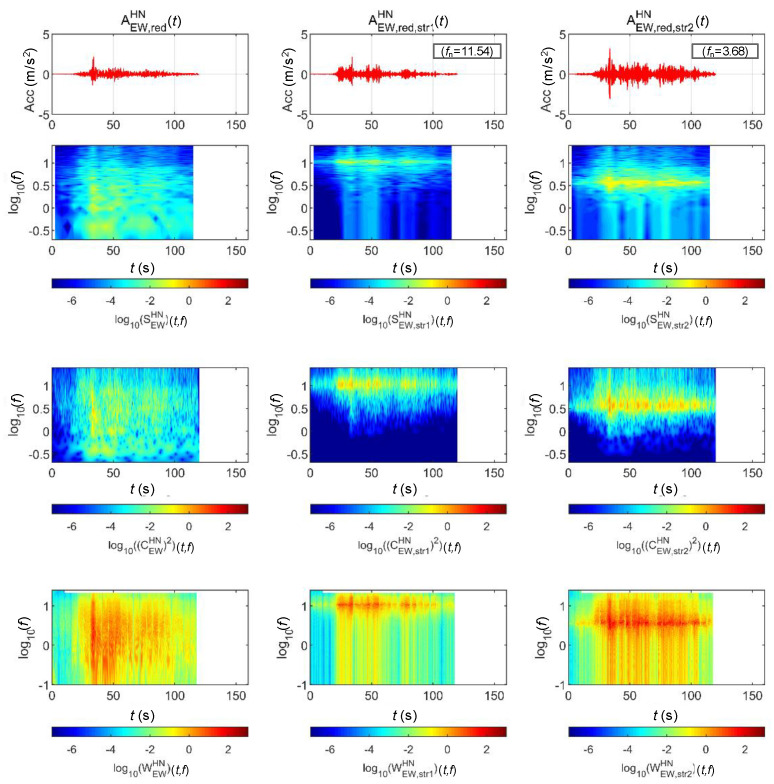
Analysis results of the Hachinohe earthquake data (scaled for 2400-year return period) and associated bridge response.

Figure 17 and Figure 18 show the STFT, WT, and WVD results for the accelerations and bridge responses of the Hachinohe earthquake adjusted to return periods of 1000 and 2400 years, respectively. Unlike the Gyeongju and Pohang earthquakes, the Hachinohe earthquake showed a certain level of energy in the 0.3–10 Hz (10^−0.5^–10^1^) region, even after 30 s, when the peak acceleration occurred. This is similar to the spectral density result shown in Figure 4. An examination of bridge responses shows the formation of high energy near the natural frequencies of both BR2 and BR3 bridges. Finally, the analysis results of the responses in the acceleration of the Hachinohe earthquake indicate that the WVD method had the most dynamic result of energy change, compared with STFT and WT.

#### 3.2.2. Train Data

**Figure 19:**
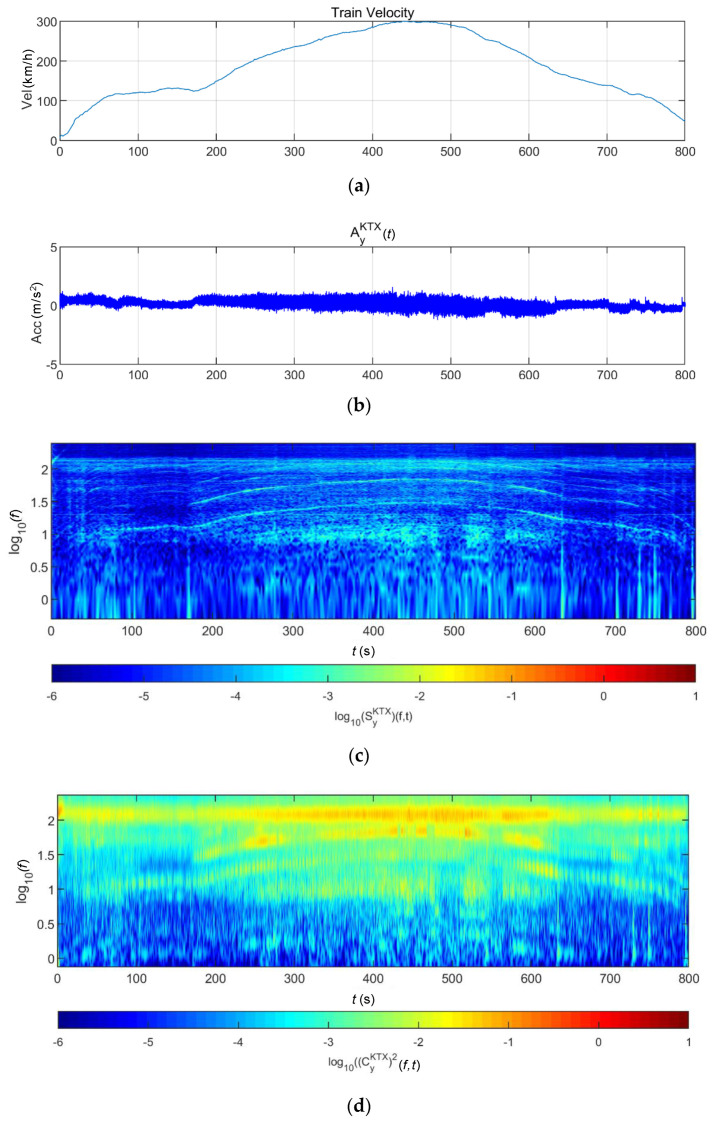

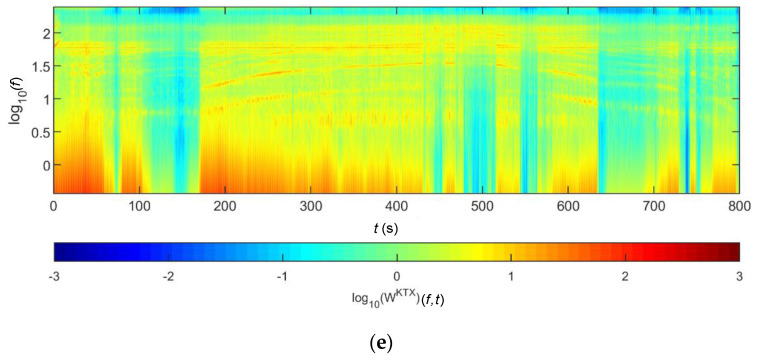
Analysis results of train data: (**a**) velocity; (**b**) lateral acceleration time series; (**c**) STFT; (**d**) WT; (**e**) WVD.

Figure 19 shows the lateral acceleration measurement data of the train and the analysis results of STFT, WT, and WVD. STFT and WT results are the same as the results from Moon and Yoo [19]. First, high energy appeared at approximately 100 Hz for all time intervals. It is shown higher from 200 s, when the second acceleration occurred, until 600 s when the first deceleration stopped. This is the result of the train elements maintaining a certain level of rotation during operations. In comparison, in the 10–30 Hz (10^1^–10^1.5^) range, there were two line-shaped contents in which the frequency changed with the velocity. For these line-shaped contents, not only the frequency level but also the energy increased with the velocity. These line-shaped contents are believed to be related to the train elements (e.g., wheels and gears) directly associated with velocity. In the frequency range of 3 Hz (10^0.5^) and lower, high energy levels appeared during acceleration and deceleration, and low energy levels appeared when operating at a constant velocity. This suggests a relationship with velocity-change devices (e.g., accelerators and breaks). Furthermore, it can be seen that the accelerator had a larger frequency domain of generated energy and a higher energy level than did the decelerator. To examine the characteristics of each method, the STFT shows clear overall trends of data but provides low resolutions at 3 Hz and lower. This is because the *y*-axis is expressed in a log scale with the resolution of the STFT itself. The WT shows a clear trend in the high-frequency range but fails to detect low-frequency contents of 3 Hz and lower related to the acceleration and deceleration devices. Unlike the above two methods, the WVD shows a clear increase and decrease in energy in the low-frequency range of 3 Hz and lower. Furthermore, the energy-change trend in the time-frequency domain appears clearly in the range of 3 Hz and higher.

### 3.3. Data Synthesis

**Figure 20:**
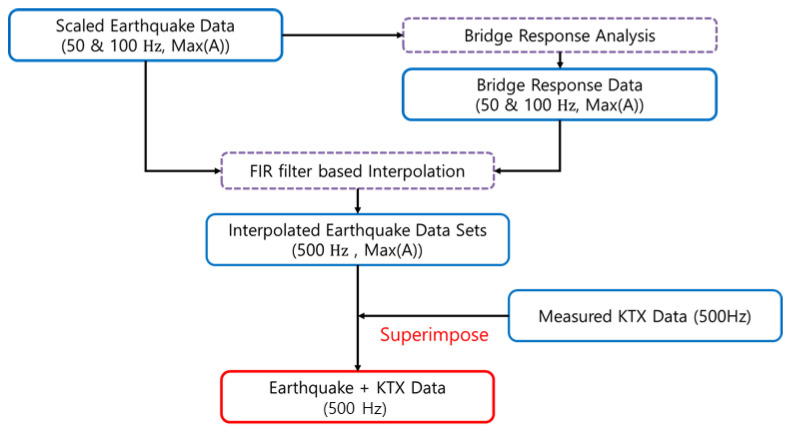
Data-synthesis flowchart.

**Figure 21:**
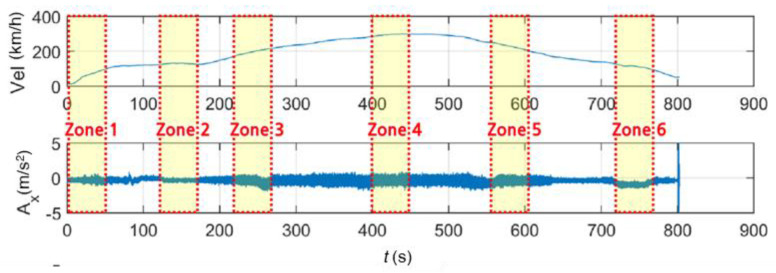
Zones selected from earthquake data superimposed with train data.

To evaluate the applicability of the above methods, virtual earthquake detection data in trains were constructed using the following process (Figure 20). First, in the case of scaled earthquake data and bridge response data, the sampling frequencies were 100 and 50 Hz, but the sampling frequency of the acceleration measurement data of the train was 500 Hz. Thus, the 100-Hz data were converted to 500 Hz via interpolation. Next, the train-measurement data during a virtual earthquake was composed via superimposition. Because the train measurement data could be changed according to the train operation conditions, as shown in Figure 21, the first acceleration zone (0–50 s, zone 1), low equal-speed zone (130–180 s, zone 2), second acceleration zone (220–270 s, zone 3), highest-speed zone (400–450 s, zone 4), first deceleration zone (550–600 s, zone 5), and second deceleration zone (720–770 s, zone 6) were selected. The initial 50-s section of each earthquake data is superimposed with train data of the above zones, respectively. This enables us to evaluate the detection of earthquakes at different train conditions. In this study, the lateral (*y*-direction) data and earthquake-related data of the train were superimposed and are directly related to the operation safety and derailment of trains.

## 4. Results and Discussion

This section provides the STFT, WT, and WVD results for the train response superimposed with 3 earthquake data sets and associated bridge response. To see the applicability of these methods to earthquake detection, results must be compared with the STFT, WT, and WVD results of train data, presented in Figure 19. Following discussion and analysis are provided based on the comparison. Some of the results presented here are the same as in the paper by Moon and Yoo [19]. These are used in this study as a comparison.

### 4.1. Train-Measured Seismic Data

**Figure 22:**
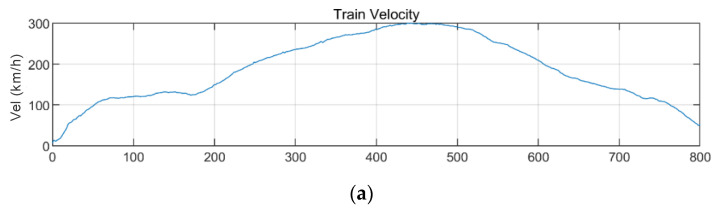

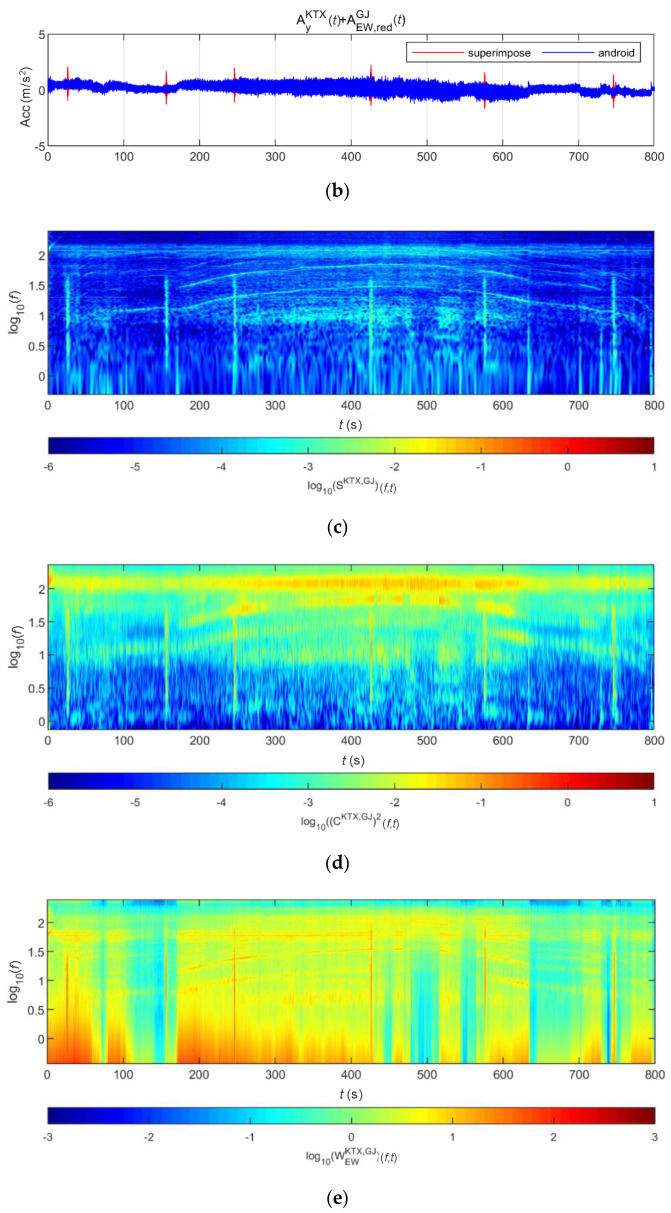
Combination of train data with the Gyeongju earthquake data (scaled to 1000-year return period): (**a**) velocity; (**b**) lateral acceleration time series; (**c**) STFT; (**d**) WT; (**e**) WVD.

**Figure 23:**
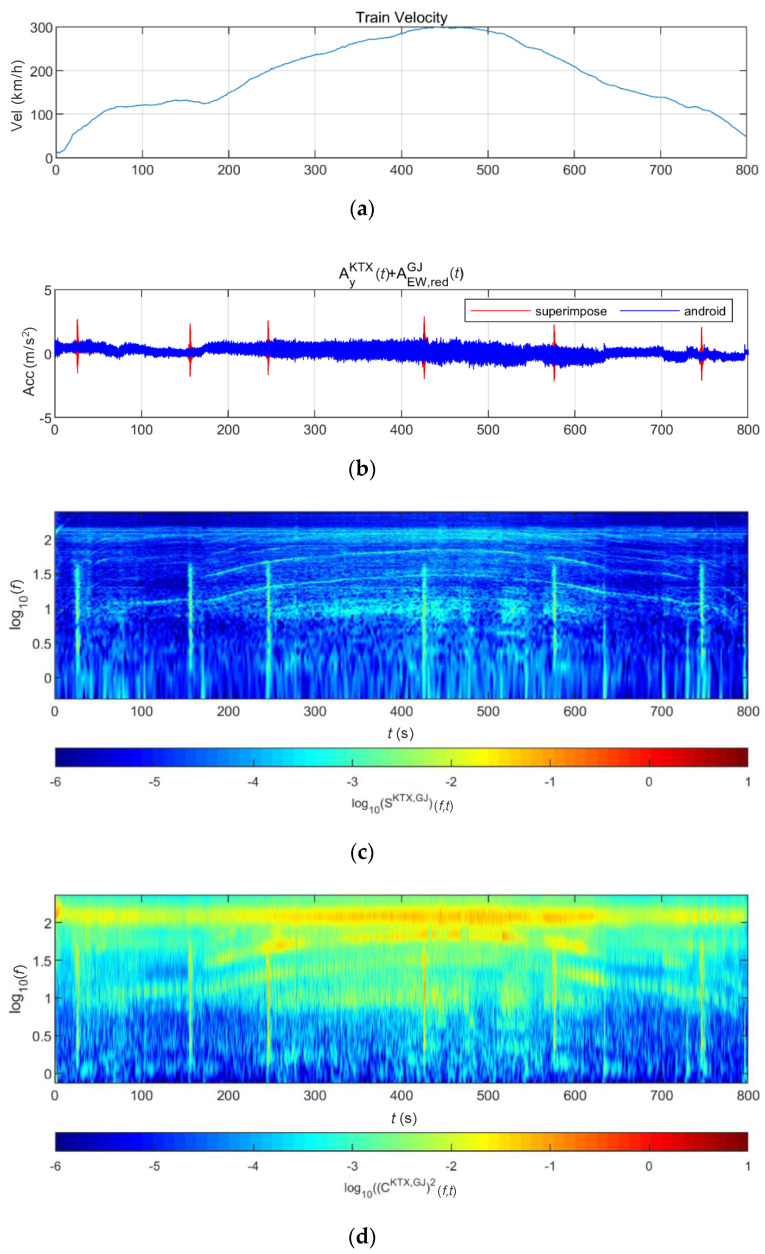

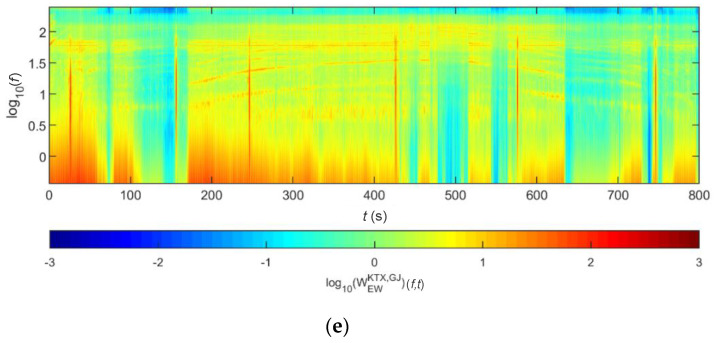
Combination of train data with the Gyeongju earthquake data (scaled to 2400-year return period): (**a**) velocity; (**b**) lateral acceleration time series; (**c**) STFT; (**d**) WT; (**e**) WVD.

Figure 22 and Figure 23 show the STFT, WT, and WVD results for the train responses superimposed with the Gyeongju earthquake data adjusted to return periods of 1000 and 2400 years, respectively. First, the STFT in Figure 22 shows clear differences in earthquake and train data at the 150-s interval where the train runs at a constant speed. In the 200–600 s interval in which the second acceleration, maximum velocity, and first deceleration occur, there is an energy difference (short period) compared with the train data. This trend appears similarly in the STFT results shown in Figure 23. The WT shows high energy compared with the existing train data in the frequency range of 3–20 Hz (10^0.5^–10^1.3^) for earthquakes having a return period of 1000 years. However, clear differences from the train data are not observed in the high-frequency ranges above 20 Hz. WT evaluated the frequency width of the train energy widely because the frequency resolution decreased in the high-frequency range. For earthquakes having a return period of 2400 years, clear differences are observed in the entire time-frequency domain as the earthquake increased. Lastly, the WVD shows clear differences in the entire time-frequency domain for both earthquakes having return periods of 1000 and 2400 years. Particularly in the acceleration time interval of the 1-Hz-or-lower frequency range of the train, energy changes caused by the earthquake clearly appear, even though the train energy was high.

**Figure 24:**
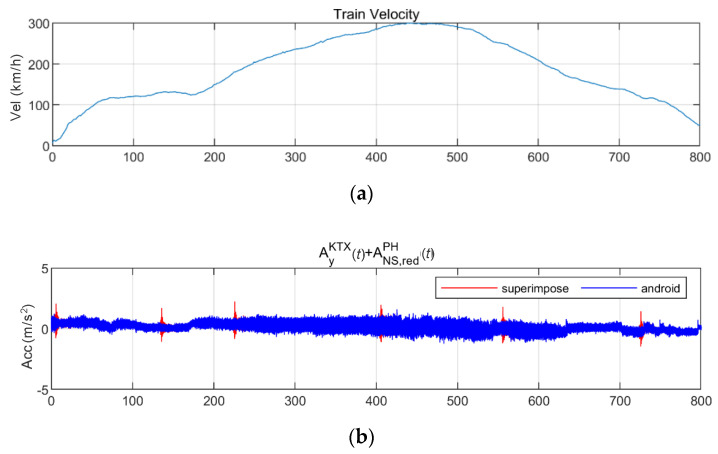

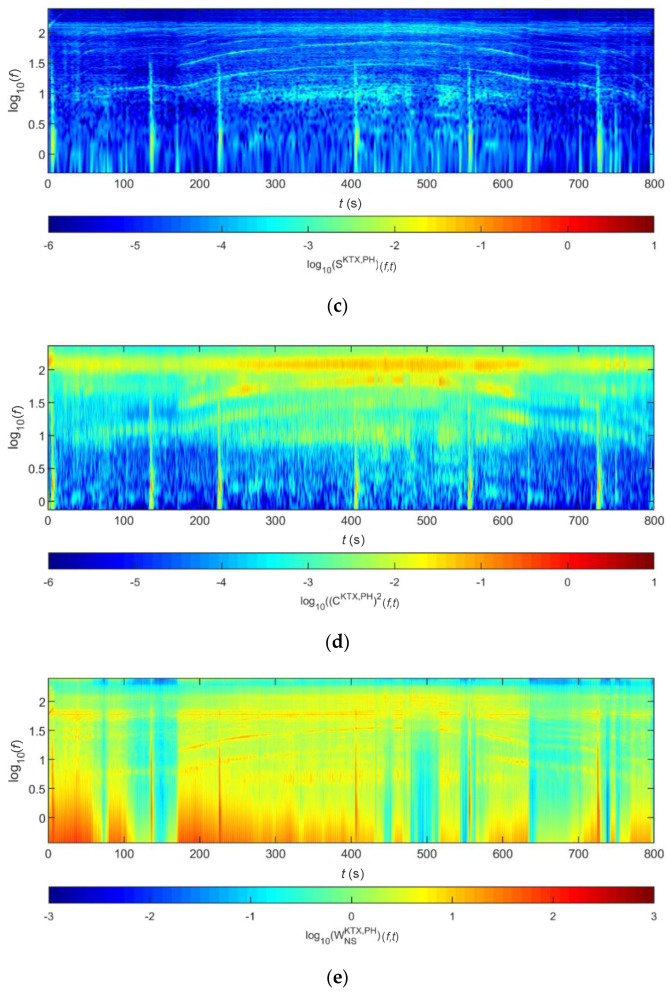
Combination of train data with the Pohang earthquake data (scaled to 1000-year return period): (**a**) velocity; (**b**) lateral acceleration time series; (**c**) STFT; (**d**) WT; (**e**) WVD.

**Figure 25:**
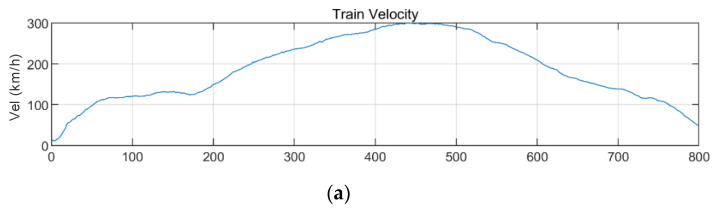

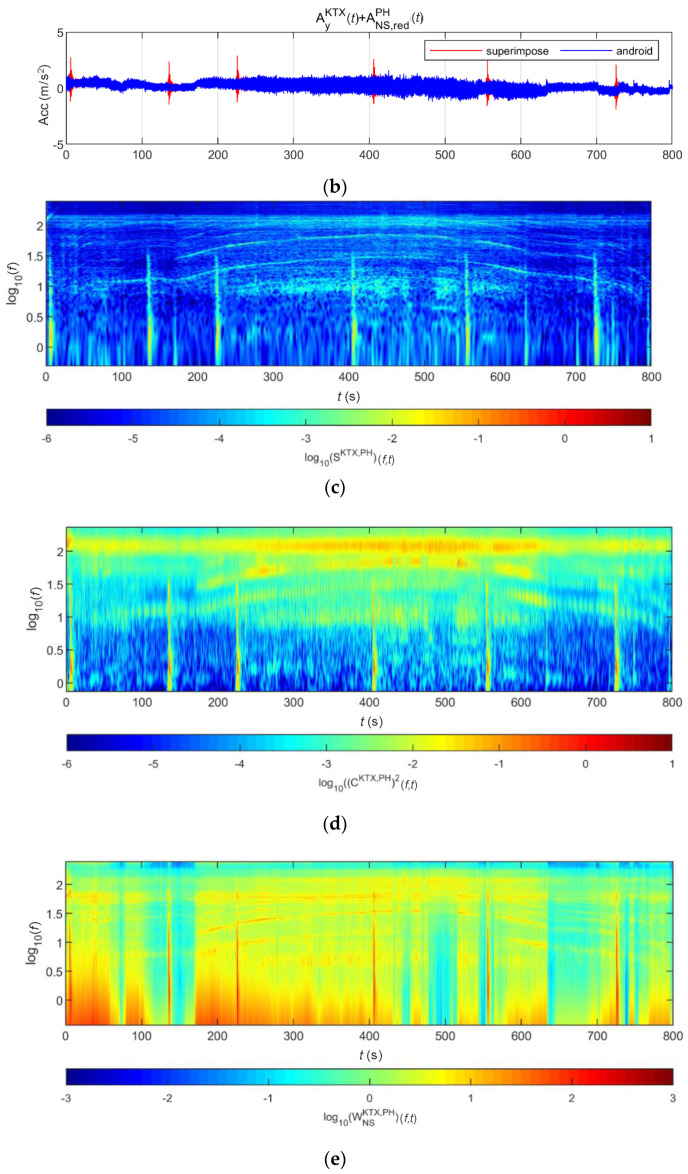
Combination of train data with the Pohang earthquake data (scaled to 2400-year return period): (**a**) velocity; (**b**) lateral acceleration time series; (**c**) STFT; (**d**) WT; (**e**) WVD.

Figure 24 and Figure 25 show the STFT, WT, and WVD results for the train responses that were superimposed with the Pohang earthquake adjusted to return periods of 1000 and 2400 years, respectively. The STFT shows clear differences in energy level compared with the train in the 10-Hz-or-lower frequency range for earthquakes having a return period of 1000 years. However, clear differences are not observed in the frequency range above 10 Hz. The WT shows high energy, compared with the existing train data in the frequency range of 10-Hz-or-lower for earthquakes having return periods of 1000 and 2400 years. However, clear differences from the train data are not observed in high-frequency ranges above 10 Hz. This is because the high-energy region of the Pohang earthquake was in a lower frequency range than that of the Gyeongju earthquake. Lastly, the WVD shows clear differences in the frequency range of 1–20 Hz (10^0^–10^1.3^) for both earthquakes having return periods of 1000 and 2400 years. Furthermore, differences are seen at the 1-Hz-or-lower frequency range of all time zones except for the first acceleration zone. The peak contents are seen red, surrounded by yellow contents, which are lower.

**Figure 26:**
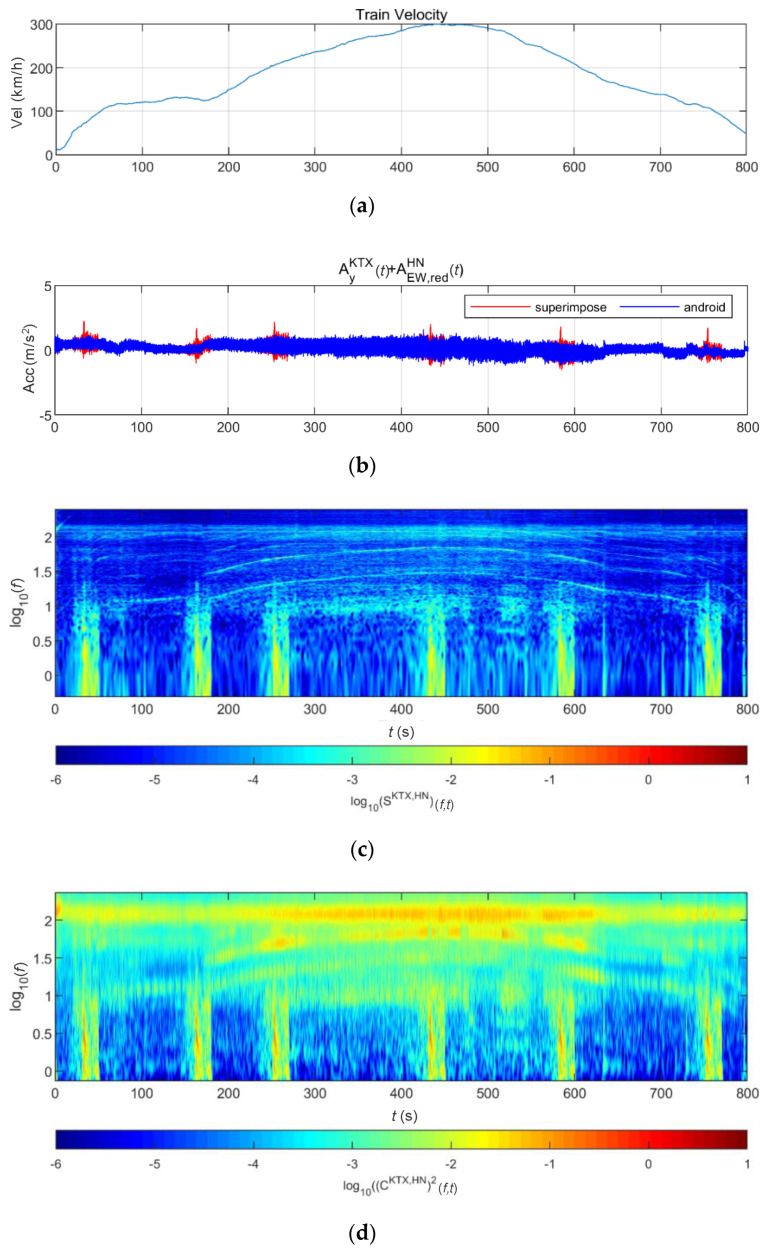

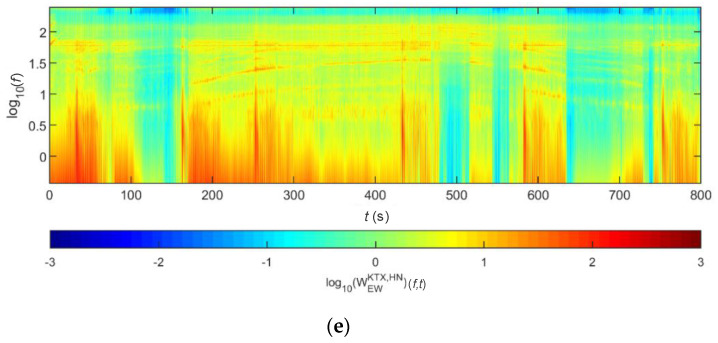
Combination of train data with the Hachinohe earthquake data (scaled to 1000-year return period): (**a**) velocity; (**b**) lateral acceleration time series; (**c**) STFT; (**d**) WT; (**e**) WVD.

**Figure 27:**
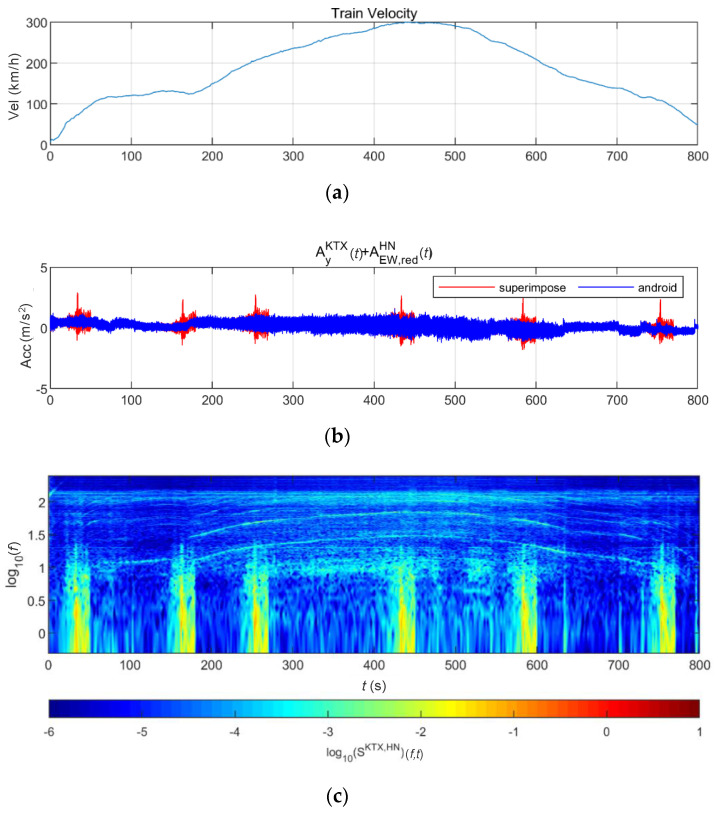

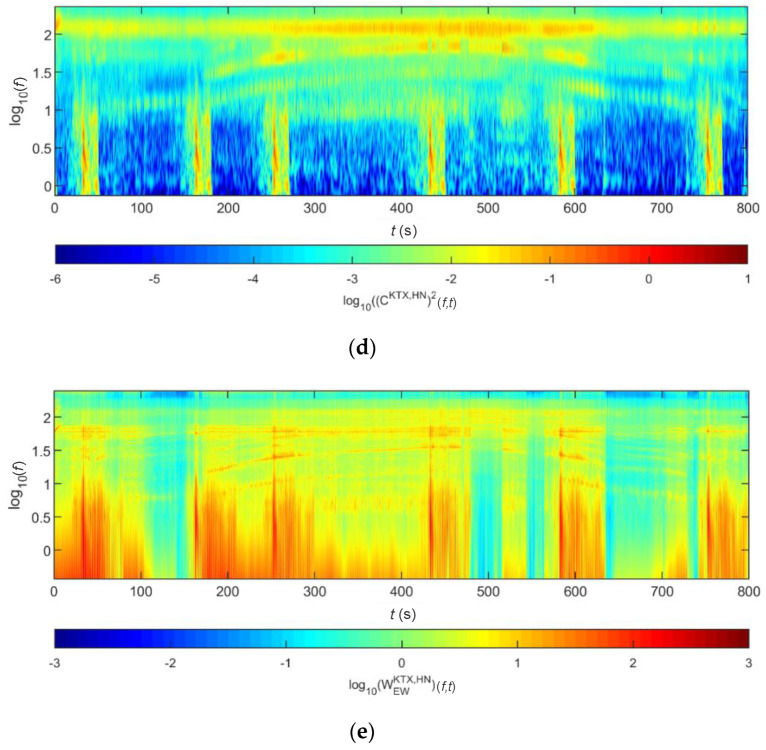
Combination of train data with the Hachinohe earthquake data (scaled to 2400-year return period): (**a**) velocity; (**b**) lateral acceleration time series; (**c**) STFT; (**d**) WT; (**e**) WVD.

Figure 26 and Figure 27 show the STFT, WT, and WVD results for the train responses that were superimposed with the Hachinohe earthquake adjusted to return periods of 1000 and 2400 years, respectively. The STFT shows clear differences for return periods of 1000 and 2400 years. The energy also shows clear differences, especially at the peak acceleration. The WT shows clear differences for return periods of 1000 and 2400 years. The STFT and WT cannot clearly detect the energy of low frequencies that occur during train acceleration, whereas they detect the energy of the Hachinohe earthquake well in a low-frequency range. The WVD clearly shows differences at peak acceleration. However, after peak acceleration, the energy of the Hachinohe earthquake was distributed over a wider frequency range than that of the energy in the low range, but the energy size does not show significant differences.

### 4.2. Train-Measured Bridge-Response Data

#### 4.2.1. BR2 Bridge

**Figure 28:**
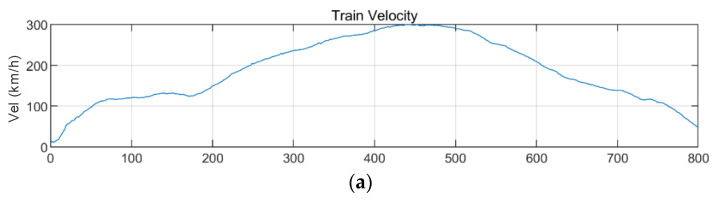

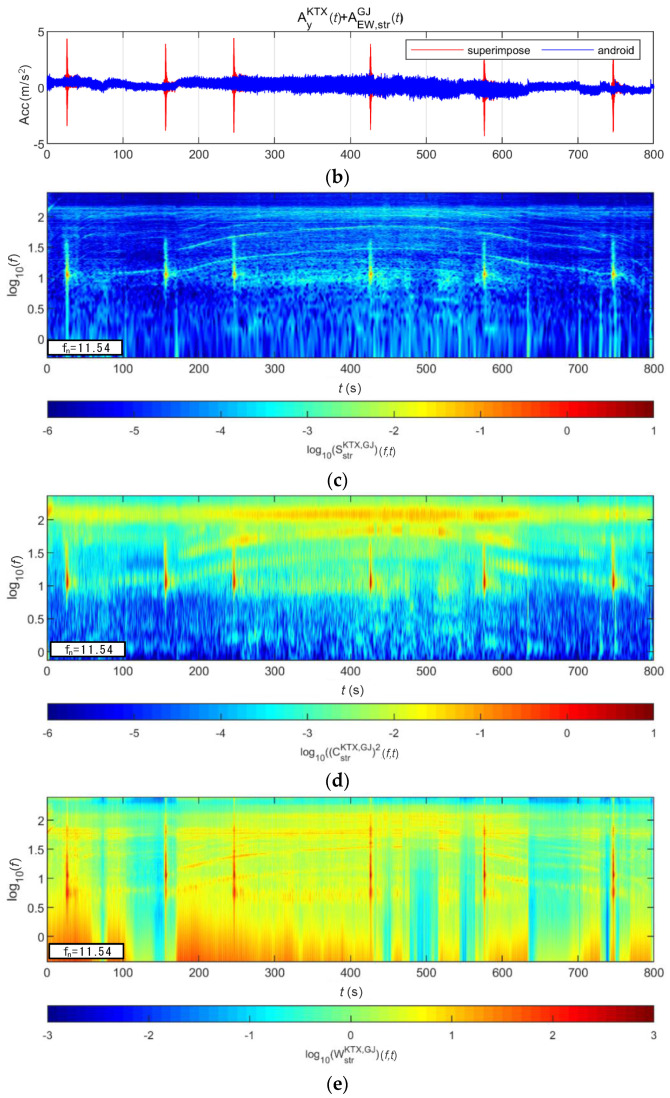
Combination of train data with BR2 bridge-response data from the Gyeongju earthquake (scaled to 1000-year return period): (**a**) velocity; (**b**) lateral acceleration time series; (**c**) STFT; (**d**) WT; (**e**) WVD.

**Figure 29:**
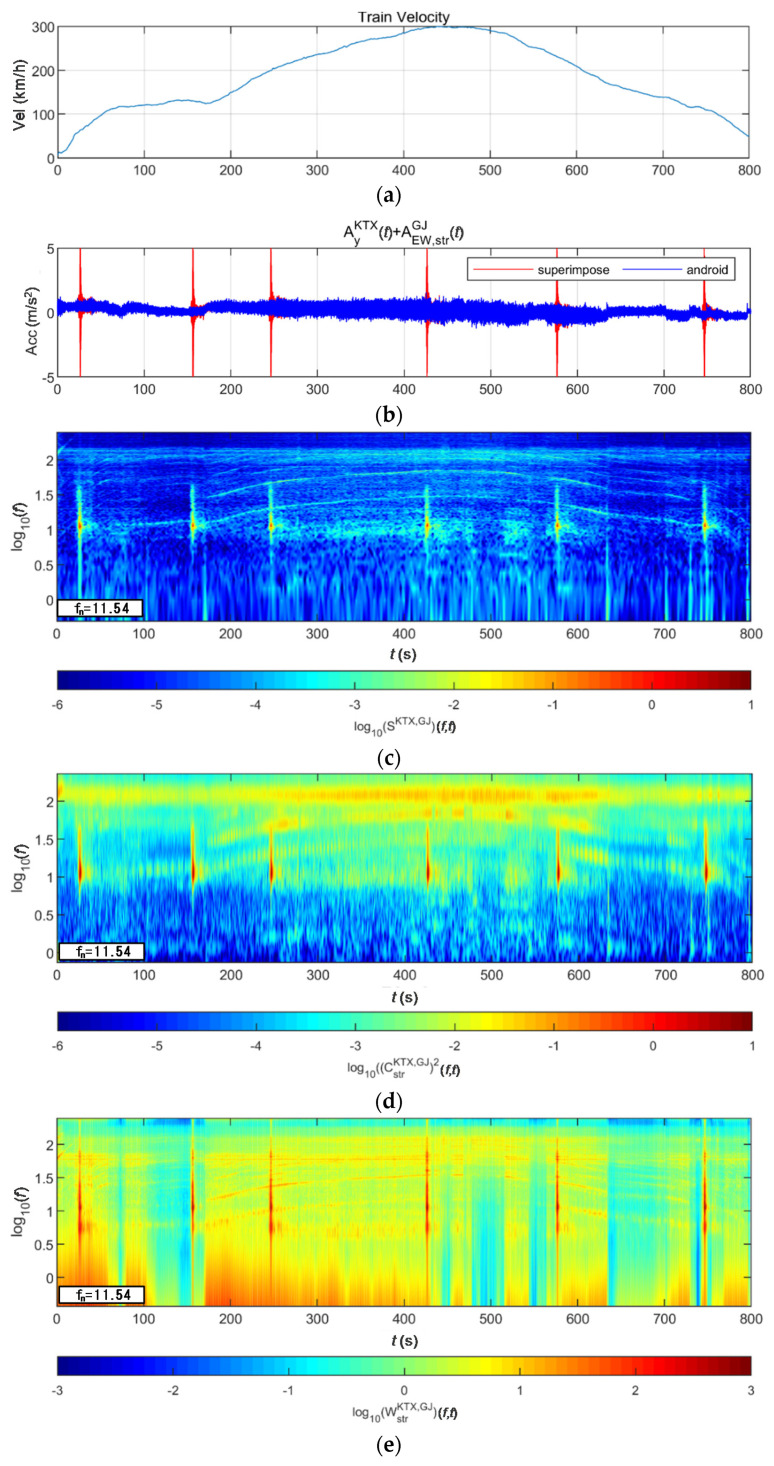
Combination of train data with BR2 bridge-response data from the Gyeongju earthquake (scaled to 2400-year return period): (**a**) velocity; (**b**) lateral acceleration time series; (**c**) STFT; (**d**) WT; (**e**) WVD.

Figure 28 and Figure 29 show the STFT, WT, and WVD results for the train responses that were superimposed with the BR2 bridge responses of the Gyeongju earthquake adjusted to return periods of 1000 and 2400 years, respectively. The STFT shows the highest energy near 11 Hz (10^1.1^) corresponding to the natural frequency of the bridge for both bridge responses with return periods of 1000 and 2400 years, which differ from the train data. The WT and WVD also show clear differences from the train data near the natural frequency of the bridge for both bridge responses having return periods of 1000 and 2400 years. In the case of the Gyeongju earthquake, the BR2 bridge response had considerable energy, confirming that the three methods were all valid. Furthermore, the WT and WVD show energy differences more vividly than does the STFT.

**Figure 30:**
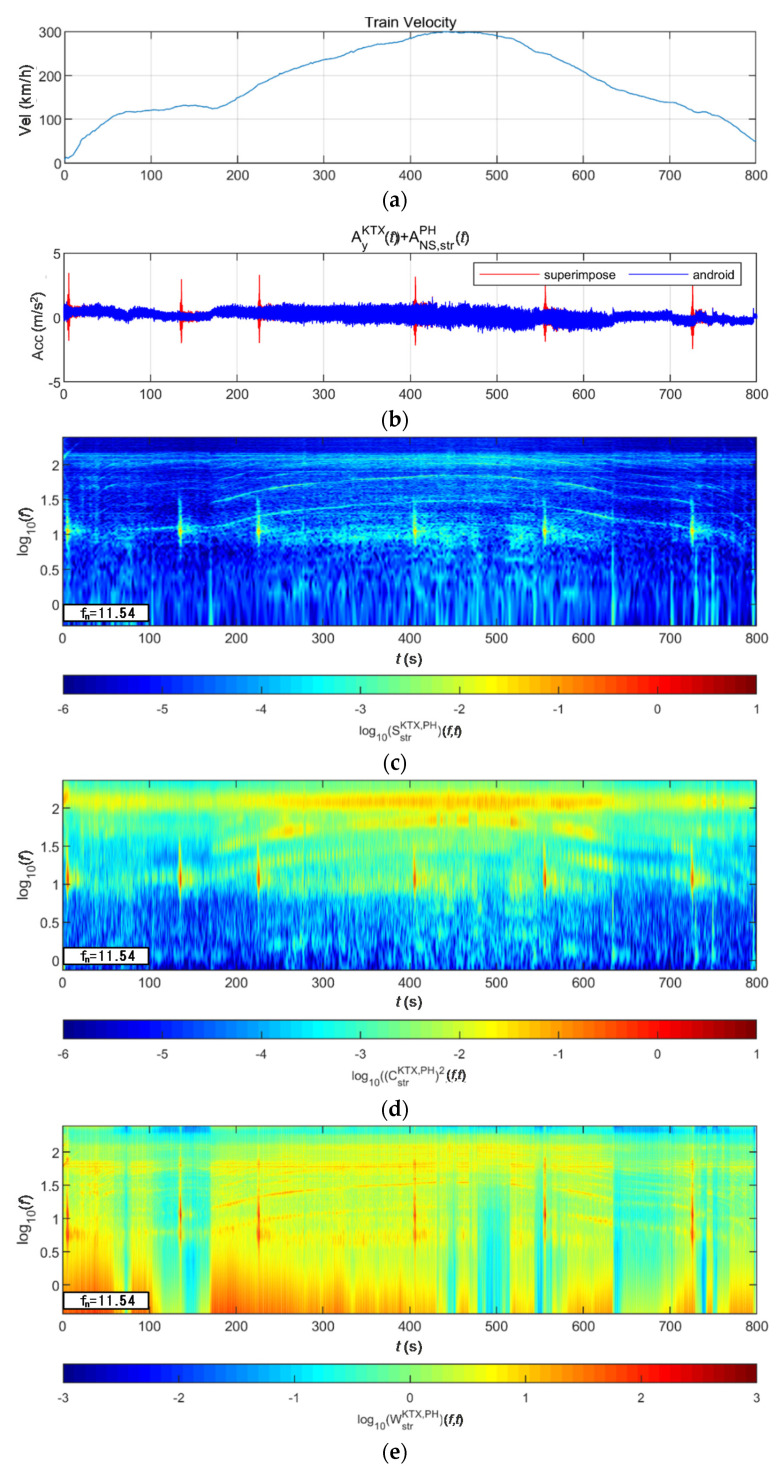
Combination of train data with BR2 bridge-response data from the Pohang earthquake (scaled to 1000-year return period): (**a**) velocity; (**b**) lateral acceleration time series; (**c**) STFT; (**d**) WT; (**e**) WVD.

**Figure 31:**
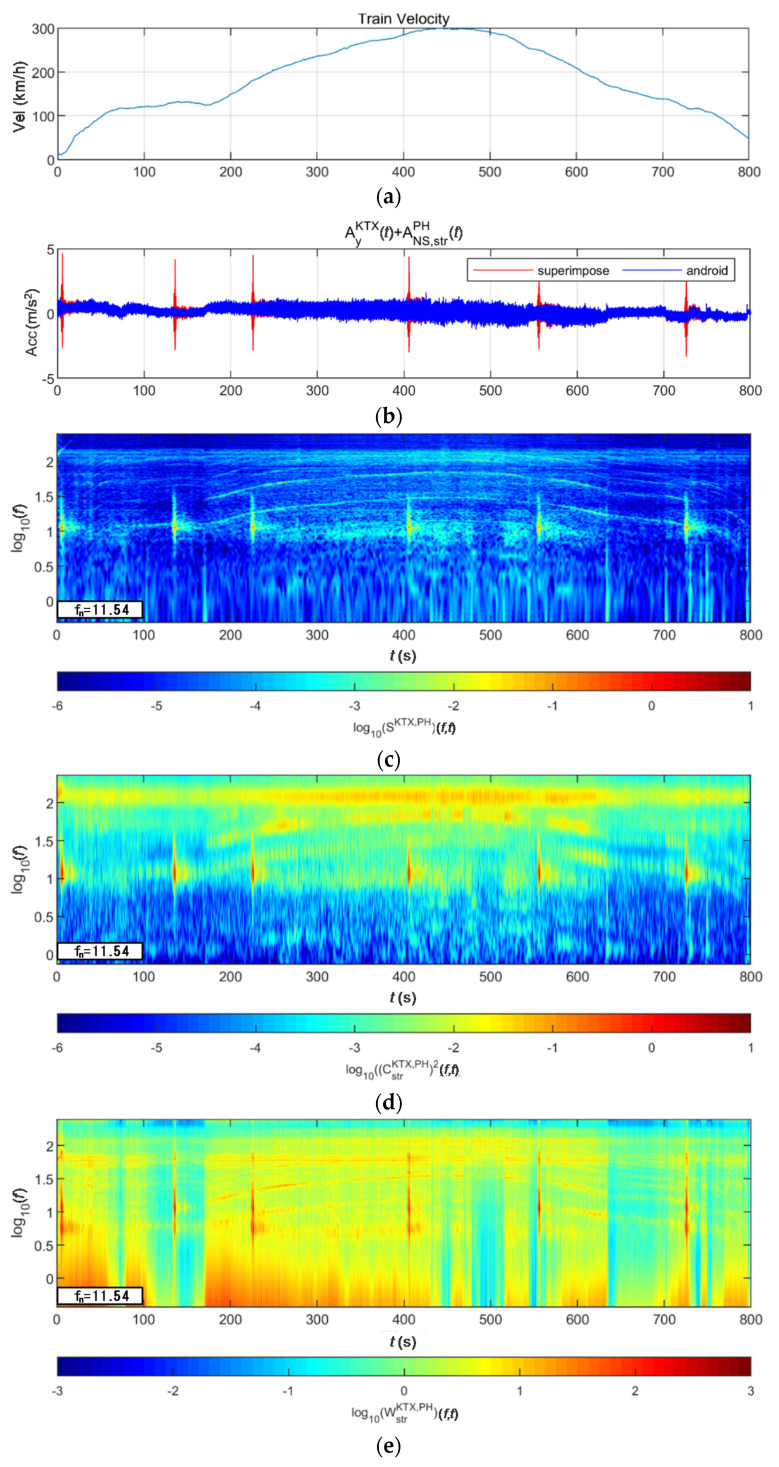
Combination of train data with BR2 bridge-response data from the Pohang earthquake (scaled to 2400-year return period): (**a**) velocity; (**b**) lateral acceleration time series; (**c**) STFT; (**d**) WT; (**e**) WVD.

Figure 30 and Figure 31 show the STFT, WT, and WVD results for the train responses that were superimposed with the BR2 bridge responses of the Pohang earthquake adjusted to return periods of 1000 and 2400 years, respectively. The STFT does not show clear differences when the bridge response having a return period of 1000 years was applied to the maximum speed section because it overlapped with the train energy. However, in other sections, the bridge response shows clear differences from the train responses. The bridge response having a return period of 2400 years shows differences, even in the maximum velocity section. The WT and WVD show clear differences in all bridge responses and velocity sections. The bridge responses by the Pohang earthquake were also detected more sensitively by the WT and WVD than by the STFT, similar to the bridge responses to the Gyeongju earthquake, described above.

**Figure 32:**
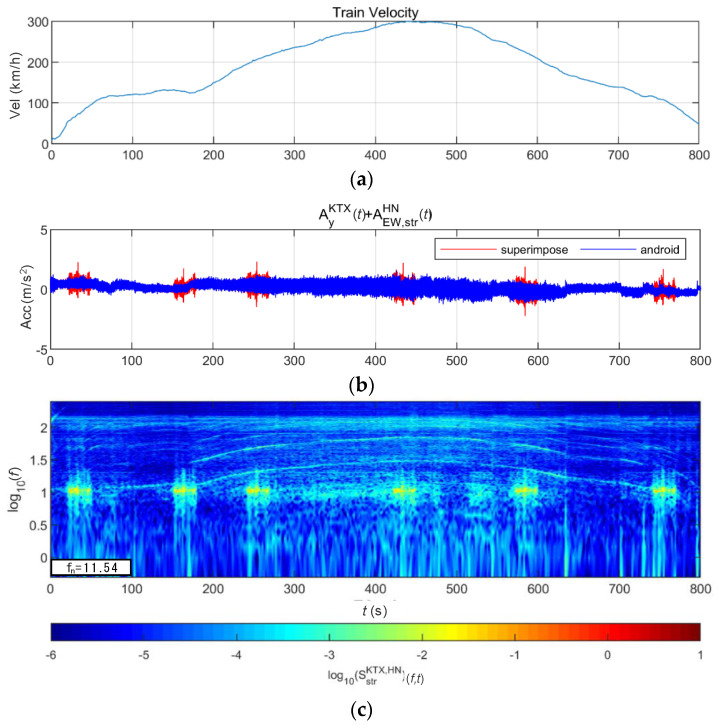

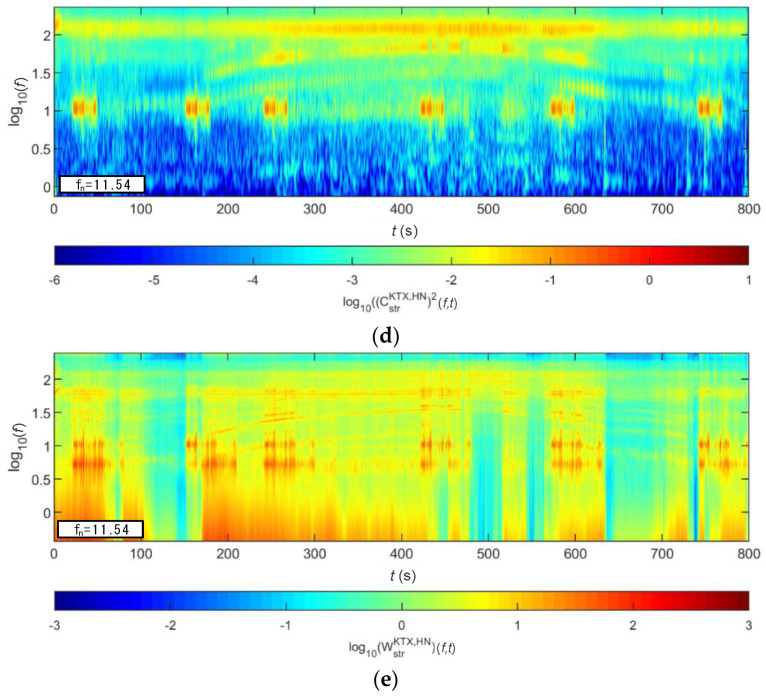
Combination of train data with BR2 bridge-response data from the Hachinohe earthquake (scaled to 1000-year return period): (**a**) velocity; (**b**) lateral acceleration time series; (**c**) STFT; (**d**) WT; (**e**) WVD.

**Figure 33:**
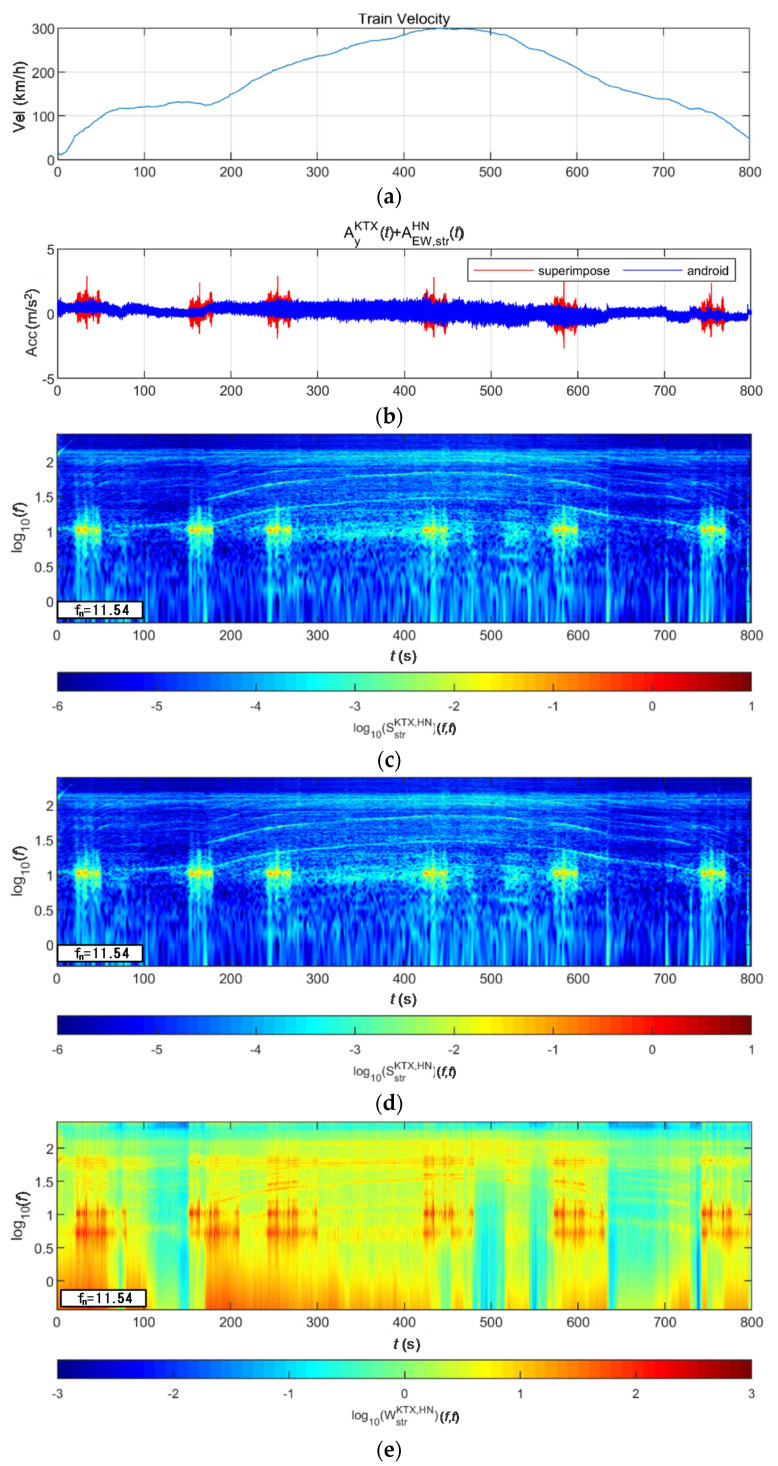
Combination of train data with BR2 bridge-response data from the Hachinohe earthquake (scaled to 2400-year return period): (**a**) velocity; (**b**) lateral acceleration time series; (**c**) STFT; (**d**) WT; (**e**) WVD.

Figure 32 and Figure 33 show the STFT, WT, and WVD results for the train responses that were superimposed with the BR2 bridge responses of the Hachinohe earthquake adjusted to return periods of 1000 and 2400 years, respectively. The STFT shows clear differences in the shape of energy distribution and energy for both bridge responses with return periods of 1000 and 2400 years. This is because, with the Hachinohe earthquake, the input seismic response was a long-period seismic wave. The WT and WVD detected bridge responses in great detail.

When the analysis result of the BR2 bridge response is examined, WVD and WT show excellent performance based on short-period earthquakes, such as those of Gyeongju and Pohang. However, it may be difficult to detect small-bridge responses. Nonetheless, all methods can sufficiently detect bridge responses generated by long-period seismic waves, including bridge response, based on the Hachinohe earthquake.

#### 4.2.2. BR3 Bridge

**Figure 34:**
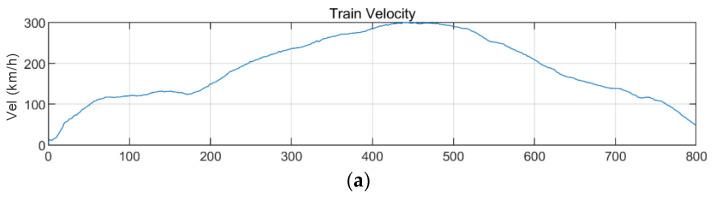

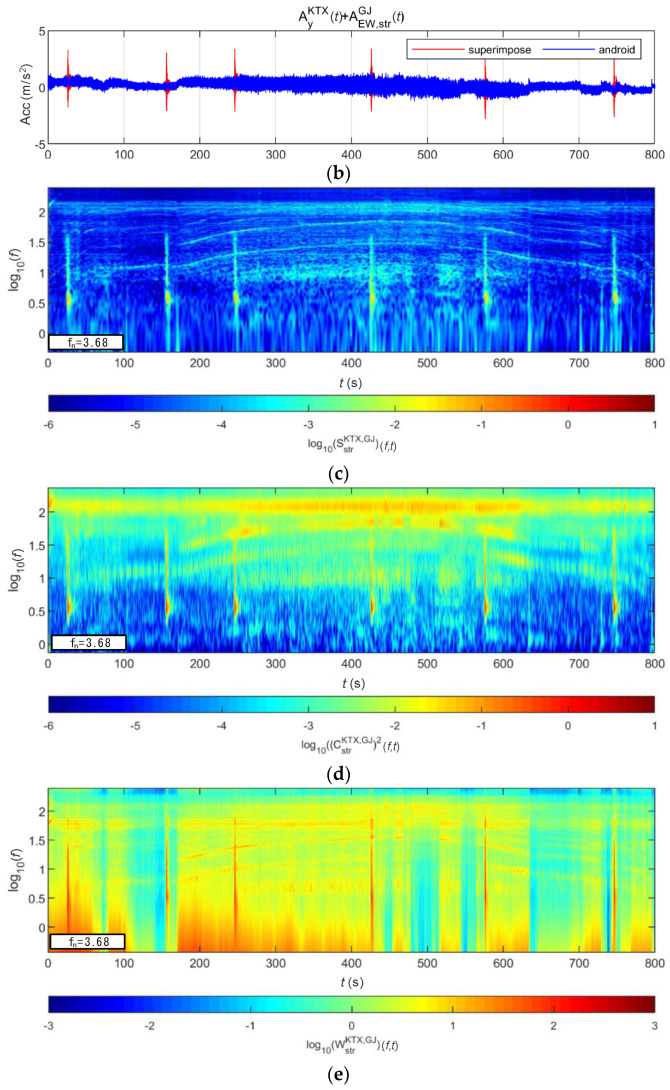
Combination of train data with BR3 bridge-response data from the Gyeongju earthquake (scaled to 1000-year return period): (**a**) velocity; (**b**) lateral acceleration time series; (**c**) STFT; (**d**) WT; (**e**) WVD.

**Figure 35:**
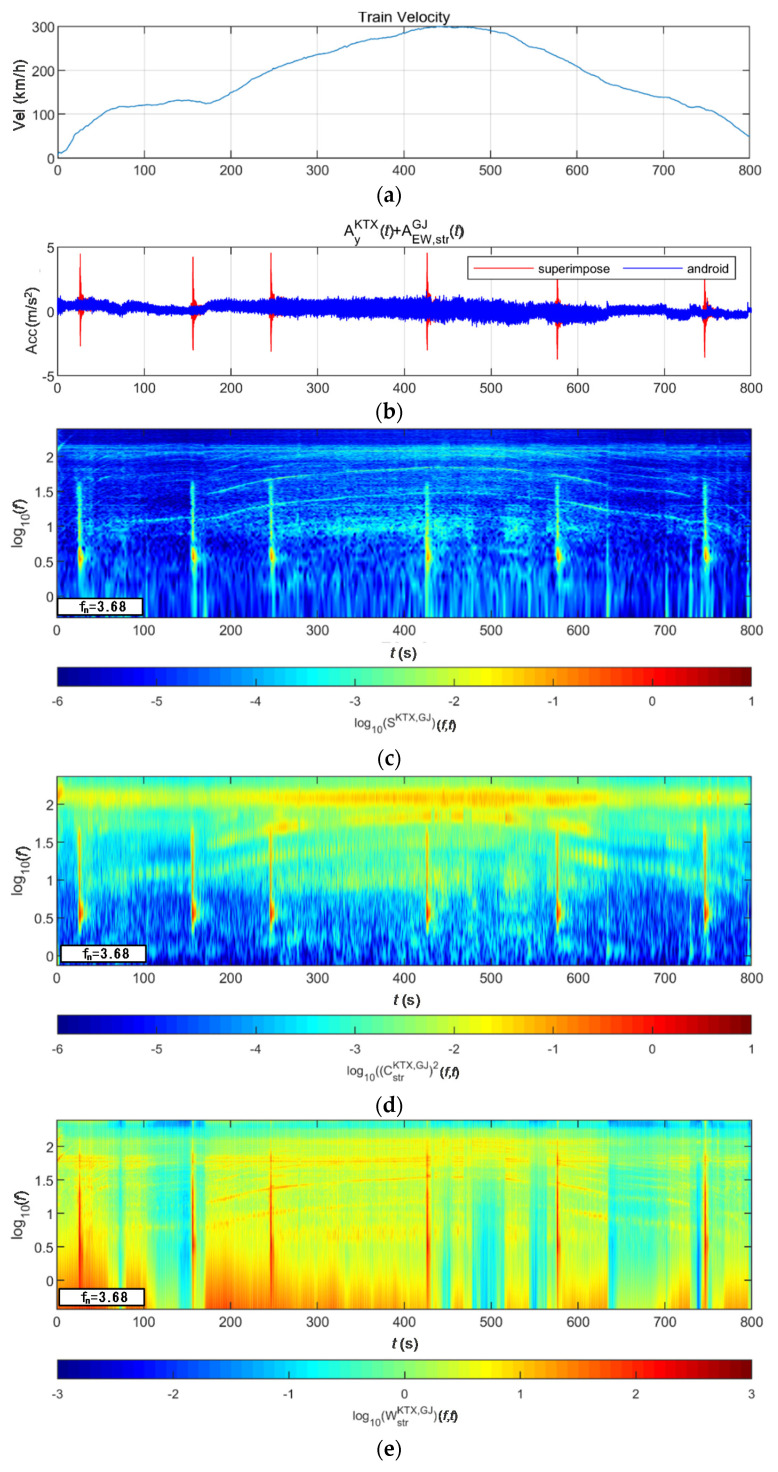
Combination of train data with BR3 bridge-response data from the Gyeongju earthquake (scaled to 2400-year return period): (**a**) velocity; (**b**) lateral acceleration time series; (**c**) STFT; (**d**) WT; (**e**) WVD.

Figure 34 and Figure 35 show the STFT, WT, and WVD results for the train responses that were superimposed with the BR3 bridge responses of the Gyeongju earthquake adjusted to return periods of 1000 and 2400 years, respectively. The STFT shows clear differences from the train data near the natural frequency of the structure. However, in the maximum-speed section, it does not show differences in the 3-Hz-or-higher frequency range. The WT shows large energy differences not only near the natural frequency of structures for both bridge responses but also in the frequency range above it. This is believed to be caused by WT characteristics, which reflect a low-frequency resolution in the high-frequency range. The WVD shows clear differences from the train data for both bridge responses.

**Figure 36:**
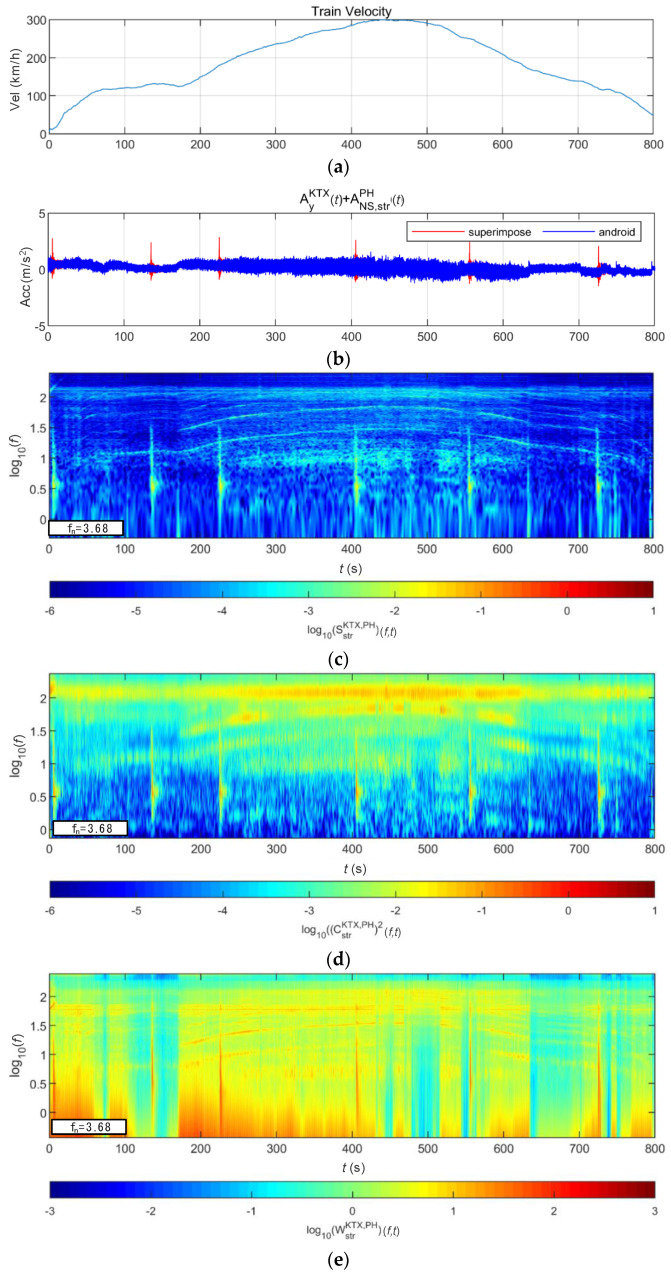
Combination of train data with BR3 bridge-response data from the Pohang earthquake (scaled to 1000-year return period): (**a**) velocity; (**b**) lateral acceleration time series; (**c**) STFT; (**d**) WT; (**e**) WVD.

**Figure 37:**
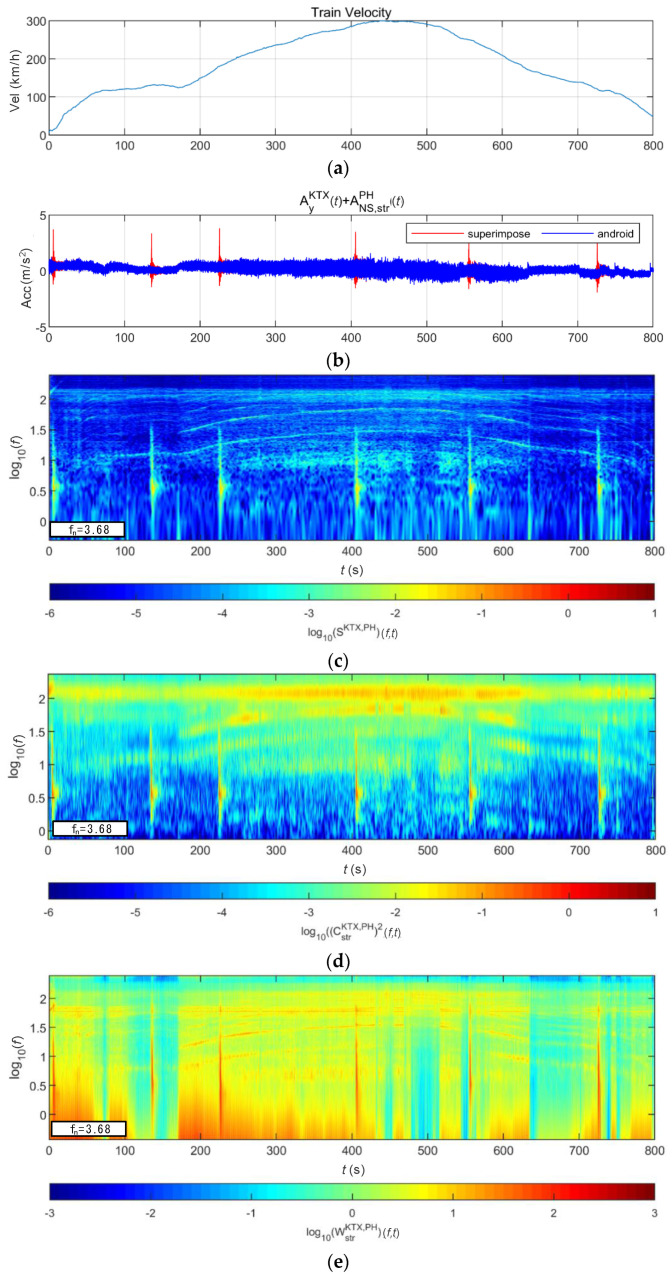
Combination of train data with BR3 bridge-response data from the Pohang earthquake (scaled to 2400-year return period): (**a**) velocity; (**b**) lateral acceleration time series; (**c**) STFT; (**d**) WT; (**e**) WVD.

Figure 36 and Figure 37 show the STFT, WT, and WVD results for the train responses that were superimposed with the BR3 bridge responses of the Pohang earthquake adjusted to return periods of 1000 and 2400 years, respectively. The STFT shows clear differences from the train data near the natural frequency of the bridge but does not show clear differences in the 4-Hz-or-higher frequency range. The WT shows similar results to the STFT for earthquake-based bridge responses in the return period of 1000 years. In the case of the bridge responses based on the return period of 2400 years, the WT method also shows clear differences over the entire frequency range. Lastly, the WVD shows clear differences from the train data in the entire time-frequency domain for both bridge responses.

**Figure 38:**
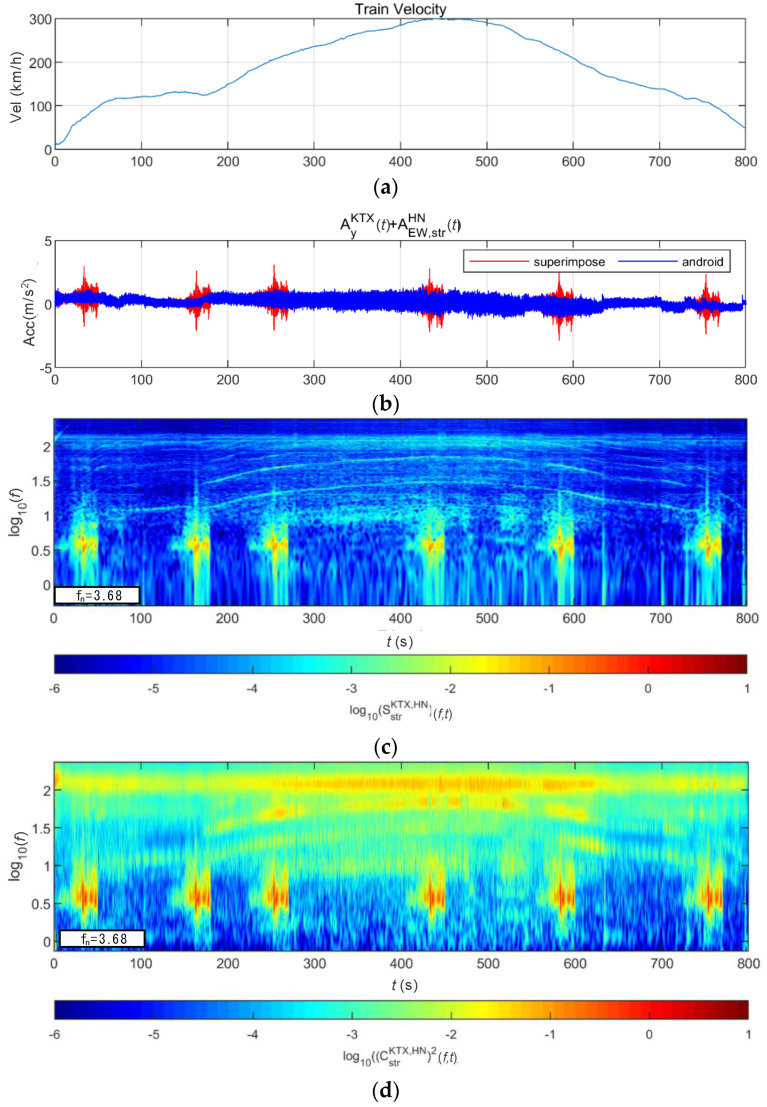

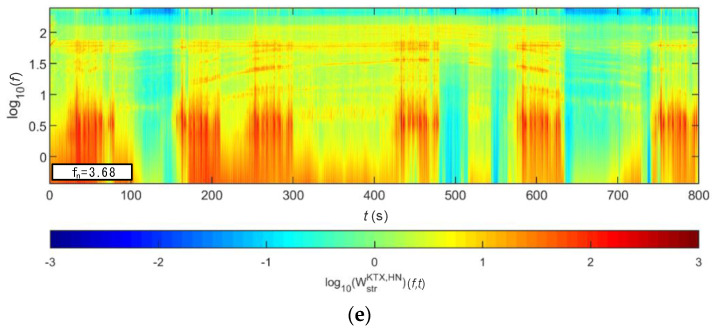
Combination of train data with BR3 bridge-response data from the Hachinohe earthquake (scaled to 1000-year return period): (**a**) velocity; (**b**) lateral acceleration time series; (**c**) STFT; (**d**) WT; (**e**) WVD.

**Figure 39:**
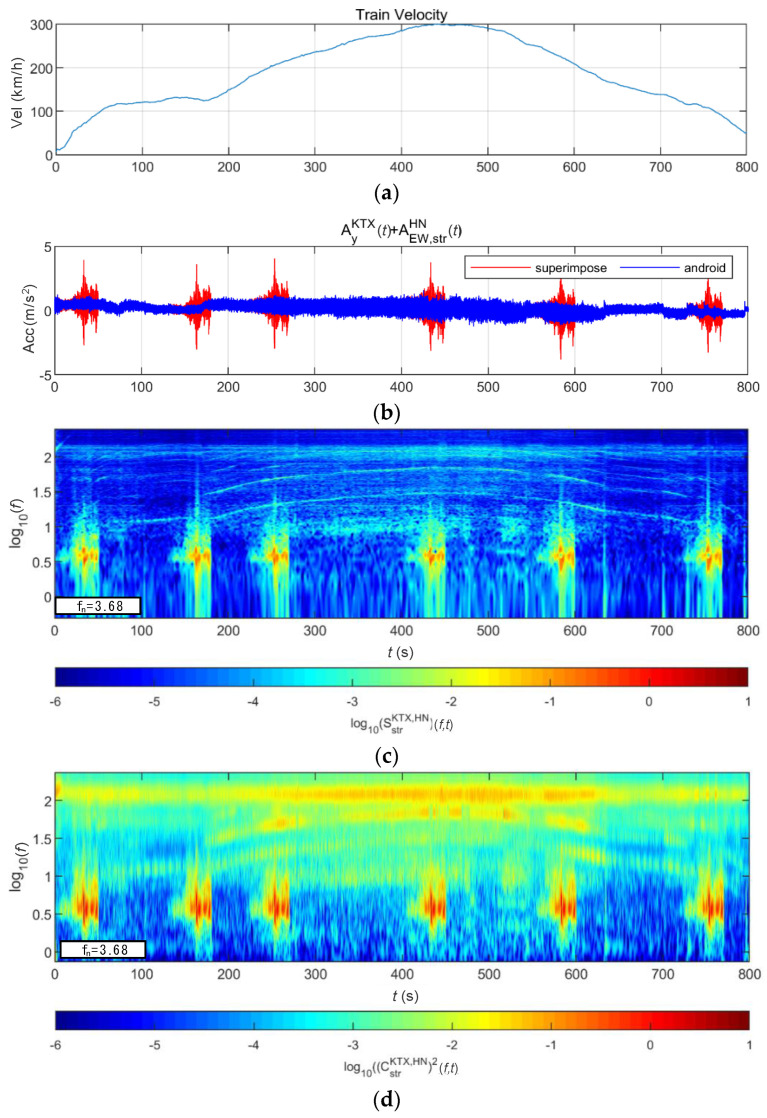

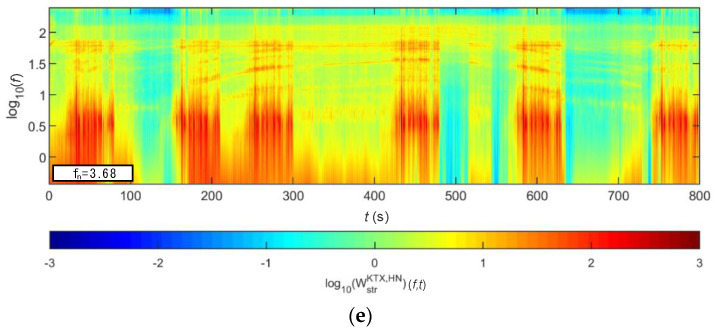
Combination of train data with BR3 bridge-response data from the Hachinohe earthquake (scaled to 2400-year return period): (**a**) velocity; (**b**) lateral acceleration time series; (**c**) STFT; (**d**) WT; (**e**) WVD.

Figure 38 and Figure 39 show the STFT, WT, and WVD results for the train responses that were superimposed with the BR3 bridge responses of the Hachinohe earthquake adjusted to return periods of 1000 and 2400 years, respectively. The STFT shows clear differences from the train data in the 1-Hz-or-lower frequency range for both ground responses. Moreover, it can be seen that owing to the nature of long-period earthquakes, the bridge responses also became long-period responses, thus increasing the expression cycle of the energy and its size. The WT also shows a similar result to that of the STFT. The WVD can sensitively detect not only the energy size but also the expansion of the time- and frequency-domain widths of energy. Thus, the WVD shows a distinct difference from the train data, although the energy of the bridge response partially overlaps the low-frequency energy of the acceleration section.

When the analysis results of the BR3 bridge response are examined, all three methods show differences in bridge responses with train data. However, if the energy of the bridge response is small, it may be difficult to show the differences with train data. The WVD method can distinguish the train data and those of the bridge-responses more easily, owing to the high resolution in the entire time-frequency domain. Furthermore, the WVD reacted to changes most sensitively.

### 4.3. Computational Time

To apply the above time-frequency analysis method to the earthquake detection and warning system, both the ability to distinguish earthquake data from train data and the computational costs required to analyze the real-time measurement data of the sensors in the trains must be considered. If onboard sensors are installed, many data sets are collected at the server. If the computational costs for the analysis are low, the server would be able to analyze data sets and provide warnings to the train promptly. However, if the computation costs for the analysis are high, the server could not able to provide warnings promptly.

To compare the computational costs among the methods, the results of performing STFT, WT, and WVD for a dataset containing 10 data points were compared. The sampling frequency of each was 500 Hz, and the total number of sample points was 16,384 (=2^14^, 32.768 s). For application to a real-time detection system, 1.025 s (2^9^ samples) was set as the window size, and FFT (STFT), WT, and WVD were applied to each using a MATLAB 2019. The processor and memory of the computer used for this calculation included an Intel ^®^ Core ™ i7-7700 HQ processor @ 2.81 GHz and 16 GB, respectively.

Table 3 shows the time required for each method. It can be seen that WVD consumes the largest calculation time. This is similar to the findings of other studies [46,47]. The WT also requires a considerable amount of time. Because the result reflects the calculation of a set of 10 data points, it can be seen that the time required for calculating one datum (1/10 of Table 3) is less than the time required to calculate the data for all three methods. However, the server would have to calculate many measured datasets which the required computational costs would be vastly different depending on the method. When the WT and WVD methods are applied to the system based on the current programming code, it becomes necessary to optimize the computational capacity, programming code, and parameters of the server for rapid detection.

## 5. Conclusions

This study evaluated time-frequency analysis methods for in-train earthquake observation data to detect earthquakes from moving trains. Android sensors were installed in a train, and the accelerations were measured during train operations. Then, the lateral responses of bridges were calculated in response to earthquakes of various periods measured inside and outside Korea. The seismic accelerations and associated bridge responses were superimposed with the train acceleration data to generate virtual earthquake observation data in the train. Virtual earthquake detection data was analyzed in the time-frequency domain by applying the STFT, WT, and WVD methods. The results of the time-frequency domain showed that the short- and long-period earthquakes were detected most sensitively by the WVD, which showed a relatively high detection ability, compared with the WT and STFT. STFT could have limits on providing clear differences if the energy of the earthquake is small and the period of an earthquake is long. CWT could have limits on providing difference if a short period earthquake with small energy occurs. Compared to others, WVD would be able to provide clear differences in all train operation conditions with various periods of earthquakes occur. Even when the train is in the first acceleration zone, where low-frequency contents are relatively high, WVD could distinguish the earthquake data at low-frequency bands. In the case of bridge responses, all three methods showed good detection ability as the energy of the response increased at the natural frequency of the bridge. The WVD showed the highest detection ability. In specific, WVD could provide meaningful results even on earthquake scaled down to 0.154 g. However, the WVD required more computational resources than did those of WT and STFT methods.

The final goal of this research project is to develop an earthquake warning system by image machine learning technique. First, the analysis results of train data with various routes are trained. Although routes are different, the analysis results would provide similar characteristics regarding the train velocity since the same train model would be operated. The figures in this study are directly used as training material. Next, the analysis results of train data superimposed with earthquake data are going to be trained to the model. By this 2-step training process, the model which detects earthquakes from the train data based on analysis figures will be constructed. The model would be able to distinguish different/singular features from analysis figures. As the server collects measured data sets and generates figures of data analysis, the model will check these figures and detect the occurrence of earthquakes. The analysis results in this study will be used as train data sets for the construction of the detection model.

The limitations of this study are as follows. First, the bridge was modeled as a 1-DoF linear model and the stiffness of the bridge pier has been accounted for. In the case of real railroad bridges, actual lateral behaviors would be much more complex. For example, the effect of the bearing, which could affect the lateral stiffness of the bridge, has not been accounted for in this study. Also, if extreme loads have been applied, such as earthquakes, the bridge must behave nonlinearly. Second, this study assumed that the response of the roadbed could be measured directly by the sensors in the train. However, the roadbed, wheels, and train tops were not completely fixed to each other. If they are not fixed, the resultant response would include multiple peak contents at different frequency ranges. Also, the response would include the friction between the track and the flange of train wheels. Third, the seismic responses could be changed according to the characteristics of other structures, including the embarkment, tunnel, and roadbed conditions. Lastly, to develop an earthquake detection and warning system, which was the objective of this study, the analysis method must be able to detect earthquakes with relatively small computational costs. Although the WVD had the highest detection ability, it incurred high computational costs.

Therefore, the following items are planned for future study. First, the nonlinearity of bridges will be considered for the earthquake response calculation. Also, a bridge model with multiple DoF will be applied to describe the bridge response in detail. This model will also include other effects such as bearing and soil-structure interaction. Second, the characteristics of changes to the measurement data in the train from the roadbed response are going to be investigated using shaking-table and field tests. Third, the roadbeds acceleration responses for various structures will be calculated, and, based on these responses, the detection of earthquakes in trains will be investigated in a more complex manner. Besides, studies related to WVD code optimization and system parameter optimization are planned to be conducted to apply a WVD method to an earthquake detection system. Finally, studies on analyzing signals and distinguishing earthquake data when the signals are compounded by other events inside and outside the trains (e.g., driving piles near the site and human-running inside the train) are planned. All of these results would be used as materials for the model training.

## Figures and Tables

**Table 1:** Summary of earthquake events used.

Index	Location	Year	Magnitude (M_W_)	Sampling Rate (Hz)	Peak Acceleration (g)
GJ	Gyeongju	2016	5.8	100	0.346 (East–West)
PH	Pohang	2017	5.4	100	0.271 (North–South)
HN	Hachinohe	1994	7.7	50	0.317 (East–West)

**Table 2:** Summary of equivalent SDOF model.

Bridge	Index	M_eff_ (t)	L_eff_ (mm)	K_eff_ (kN/mm)	ζ (%)	f_n_ (Hz)
BR2	STR 1	689.08	10,000	3621.5	5	11.54
BR3	STR 2	846.16	20,000	452.69	5	3.68

**Table 3:** Computational costs of analysis methods for 10 datasets.

Method	STFT	WT	WVD
Time (s)	0.055	4.15	12.95

## References

[1] Korea Meteorological Administration (2016). Annual Report 2016: Korea Meteorological Administration.

[2] Korea Meteorological Administration (2017). Annual Report 2017: Korea Meteorological Administration.

[3] Sheen D.H., Park J.H., Chi H.C., Hwang E.H., Lim I.S., Seong Y.J., Pak J. (2017). The first stage of an earthquake early warning system in South Korea. Seismol. Res. Lett..

[4] Nakamura Y. UrEDAS, urgent earthquake detection and alarm system, now and future. Proceedings of the 13th World Conference on Earthquake Engineering.

[5] Ashiya K. Development of a New Early Earthquake Detection and Alarm System. https://www.rtri.or.jp/publish/qr/2002/v43_2/news1.html.

[6] Cochran E.S., Lawrence J.F., Christensen C., Jakka R.S. (2009). The quake-catcher network: Citizen science expanding seismic horizons. Seismol. Res. Lett..

[7] Cochran E.S. (2018). To catch a quake. Nat. Commun..

[8] Neighbors C., Cochran E.S., Ryan K.J., Kaiser A.E. (2017). Solving for source parameters using nested array data: A case study from the Canterbury, New Zealand earthquake sequence. Pure Appl. Geophys..

[9] Kohler M.D., Guy R., Bunn J., Massari A., Clayton R., Heaton T., Chandy K.M., Ebrahimian H., Dorn C. Community seismic network and localized earthquake situational awareness. Proceedings of the 11th U.S. National Conference on Earthquake Engineering (11NCEE).

[10] Massari A., Kohler M., Clayton R., Guy R., Heaton T., Bunn J., Chandy K.M., Demetri D. Dense building instrumentation application for city-wide structural health monitoring and resilience. Proceedings of the 16th World Conference on Earthquake Engineering (16WCEE).

[11] Clayton R.W., Heaton T., Kohler M., Chandy M., Guy R., Bunn J. (2015). Community seismic network: A dense array to sense earthquake strong motions. Seismol. Res. Lett..

[12] Faulkner M., Clayton R., Heaton T., Chandy K.M., Kohler M., Bunn J., Guy R., Liu A., Olson M., Cheng M.H. (2014). Community sense and response systems: Your phone as quake detector. Commun. ACM.

[13] Kohler M.D., Heaton T.H., Cheng M.H., Singh P. Structural health monitoring through dense instrumentation by community participants: The community seismic network and quake-catcher network. Proceedings of the 10th U.S. National Conference on Earthquake Engineering (10NCEE).

[14] Kohler M.D., Heaton T.H., Cheng M.H. The community seismic network and quake-catcher network: Enabling structural health monitoring through instrumentation by community participants. Proceedings of the SPIE Smart Structures/Non-destructive Evaluation Conference.

[15] Clayton R., Heaton T., Chandy M., Krause A., Kohler M., Bunn J., Guy R., Olson M., Faulkner M., Cheng M.H. (2011). Community seismic network. Ann. Geophys..

[16] Kong Q., Allen R.M., Schreier L. (2016). MyShake: Initial observations from a global smartphone seismic network. Geophys. Res. Lett..

[17] Kong Q., Allen R.M., Schreier L., Kwon Y.-W. (2016). MyShake: A smartphone seismic network for earthquake early warning and beyond. Sci. Adv..

[18] Allen R.M., Kong Q., Martin-Short R. (2020). The MyShake platform: A global vision for earthquake early warning. Pure Appl. Geophys..

[19] Moon J.S., Yoo M. (2020). Development of a seismic detection technology for high-speed trains using signal analysis techniques. Sensors.

[20] Midorikawa S., Miura H. (2011). Re-digitization of Strong Motion Accelerogram at Hachinohe Harbor during the 1968 Tokachi-oki, Japan Earthquake. J. Jpn. Assoc. Earthq. Eng..

[21] The Ministry of Land, Infrastructure and Transport, Korean Design Standard, KDS 17 10 00 2018. http://www.kcsc.re.kr.

[22] Newmark N.M. (1959). A method of computation for structural dynamics. J. Eng. Mech. Div..

[23] Griffin D., Lim J. (1984). Signal estimation from modified short-time Fourier transform. IEEE Trans. Acoust. Speech Signal Process..

[24] Zhong J., Huang Y. (2010). Time-frequency representation based on an adaptive short-time Fourier transform. IEEE Trans. Signal Process..

[25] Wongsaroj W., Hamdani A., Thong-Un N., Takahashi H., Kikura H. (2019). Extended short-time fourier transform for ultrasonic velocity profiler on two-phase bubbly flow using a single resonant frequency. Appl. Sci..

[26] Khan A., Ko D.K., Lim S.C., Kim H.S. (2019). Structural vibration-based classification and prediction of delamination in smart composite laminates using deep learning neural network. Compos. Part B Eng..

[27] Pan X., Cheng Z., Zheng Z., Zhang Y. (2019). Sparse Bayesian learning beamforming combined with short-time Fourier transform for fault detection of wind turbine blades. J. Acoust. Soc. Am..

[28] Lei J., Liu C., Jiang D. (2019). Fault diagnosis of wind turbine based on Long Short-term memory networks. Renew. Energy.

[29] Sinha S., Routh P.S., Anno P.D., Castagna J.P. (2005). Spectral decomposition of seismic data with continuous-wavelet transform. Geophysics.

[30] Liu W., Cao S., Chen Y. (2015). Seismic time-frequency analysis via empirical wavelet transform. IEEE Geosci. Remote Sens. Lett..

[31] Anvari R., Siahsar M.A.N., Gholtashi S., Kahoo A.R., Mohammadi M. (2017). Seismic random noise attenuation using synchrosqueezed wavelet transform and low-rank signal matrix approximation. IEEE Trans. on Geosci. Remote Sens..

[32] Ukil A., Živanović R. (2006). Abrupt change detection in power system fault analysis using adaptive whitening filter and wavelet transform. Electr. Power Syst. Res..

[33] Zhang H.Q., Yan Y. (2001). A wavelet-based approach to abrupt fault detection and diagnosis of sensors. IEEE Trans. Instrum. Meas..

[34] Zhang C., Wang H., Zeng J., Ma L., Guan L. (2020). Short-term dynamic radar quantitative precipitation estimation based on wavelet transform and support vector machine. J. Meteorol. Res..

[35] Wigner E. (1932). On the quantum correction for thermodynamic equilibrium. Phys. Rev..

[36] Huerta-Lopez C.I., Shin Y., Powers E.J., Roesset J.M. Time-Frequency Analysis of Earthquake records. Proceedings of the 12th World Conference on Earthquake Engineering.

[37] Kalra M., Kumar S., Das B. (2020). Moving ground target detection with seismic signal using smooth pseudo Wigner–Ville distribution. IEEE Trans. Instrum. Meas..

[38] Wu X., Liu T. (2009). Spectral decomposition of seismic data with reassigned smoothed pseudo Wigner–Ville distribution. J. Appl. Geophys..

[39] Shekar B., Nanda D. Seismic spectral decomposition with smoothed pseudo Wigner-Ville distribution. Proceedings of the 80th EAGE Conference and Exhibition 2018, European Association of Geoscientists & Engineers.

[40] Staszewski W.J., Worden K., Tomlinson G.R. (1997). Time-frequency analysis in gearbox fault detection using the Wigner–Ville distribution and pattern recognition. Mech. Syst. Signal Process..

[41] Shin Y.S., Jeon J.J. (1993). Pseudo Wigner-Ville time-frequency distribution and its application to machinery condition monitoring. Shock Vib..

[42] Pukhova V.M., Kustov T.V., Ferrini G. Time-frequency analysis of non-stationary signals. Proceedings of the 2018 IEEE Conference of Russian Young Researchers in Electrical and Electronic Engineering (EIConRus).

[43] Tang B., Liu W., Song T. (2010). Wind turbine fault diagnosis based on Morlet wavelet transformation and Wigner-Ville distribution. Renew. Energy.

[44] Chi P.J., Russell C.T. (2008). Use of the Wigner-Ville distribution in interpreting and identifying ULF waves in triaxial magnetic records. J. Geophys. Res. Space Phys..

[45] Xu C., Wang C., Liu W. (2016). Nonstationary vibration signal analysis using wavelet-based time-frequency filter and Wigner–Ville distribution. ASME J. Vib. Acoust..

[46] Taebi A., Mansy H.A. Analysis of seismocardiographic signals using polynomial chirplet transform and smoothed pseudo Wigner-Ville distribution. Proceedings of the 2017 IEEE Signal Processing in Medicine and Biology Symposium (SPMB).

[47] Bouchikhi E.H., Choqueuse V., Benbouzid M.E.H., Charpentier J.F., Barakat G. A comparative study of time-frequency representations for fault detection in wind turbine. Proceedings of the 37th Annual Conference of the IEEE Industrial Electronics Society (IECON 2011).

[48] Fedotenkova M. (2016). Extraction de Composants Multivariés des Signaux Cérébraux Obtenus Pendant L’anesthésie Générale. Ph.D Thesis.

[49] Auger F., Flandrin P., Gonçalvès P., Lemoine O. (1995). Time-Frequency Toolbox For Use with MATLAB.

